# The role of tyrosine hydroxylase–dopamine pathway in Parkinson’s disease pathogenesis

**DOI:** 10.1007/s00018-022-04574-x

**Published:** 2022-11-21

**Authors:** Zhi Dong Zhou, Wuan Ting Saw, Patrick Ghim Hoe Ho, Zhi Wei Zhang, Li Zeng, Ya Yin Chang, Alfred Xu Yang Sun, Dong Rui Ma, Hong Yan Wang, Lei Zhou, Kah Leong Lim, Eng-King Tan

**Affiliations:** 1 National Neuroscience Institute, 11 Jalan Tan Tock Seng, Singapore, 308433 Singapore; 2 Department of Neurology, Singapore General Hospital, National Neuroscience Institute, Outram Road, Singapore, 169608 Singapore; 3 Duke-NUS Graduate Medical School, Signature Research Program in Neuroscience and Behavioural Disorders, 8 College Road, Singapore, 169857 Singapore; 4 Ocular Proteomics Laboratory, Singapore Eye Research Institute, Singapore, 169856 Singapore; 5 Department of Ophthalmology, Yong Loo Lin School of Medicine, National University of Singapore, Singapore, 119077 Singapore; 6 Ophthalmology and Visual Sciences Academic Clinical Research Program, Duke-NUS Medical School, Singapore, 169857 Singapore; 7 Developmental of Stem Cell Biology and Regenerative Medicine, Lee Kong Chian School of Medicine, Nanyang Technological University, 11 Mandalay Road, Singapore, 308232 Singapore

**Keywords:** Dopamine, LRRK2, Neurodegeneration, Parkinson’s disease, PINK1, Tyrosine hydroxylase

## Abstract

**Background:**

Parkinson’s disease (PD) is characterized by selective and progressive dopamine (DA) neuron loss in the substantia nigra and other brain regions, with the presence of Lewy body formation. Most PD cases are sporadic, whereas monogenic forms of PD have been linked to multiple genes, including *Leucine kinase repeat 2* (*LRRK2)* and *PTEN-induced kinase 1 (PINK1),* two protein kinase genes involved in multiple signaling pathways. There is increasing evidence to suggest that endogenous DA and DA-dependent neurodegeneration have a pathophysiologic role in sporadic and familial PD.

**Methods:**

We generated patient-derived dopaminergic neurons and human midbrain-like organoids (hMLOs), transgenic (TG) mouse and *Drosophila* models, expressing both mutant and wild-type (WT) LRRK2 and PINK1. Using these models, we examined the effect of LRRK2 and PINK1 on tyrosine hydroxylase (TH)–DA pathway.

**Results:**

We demonstrated that PD-linked LRRK2 mutations were able to modulate TH–DA pathway, resulting in up-regulation of DA early in the disease which subsequently led to neurodegeneration. The LRRK2-induced DA toxicity and degeneration were abrogated by wild-type (WT) PINK1 (but not PINK1 mutations), and early treatment with a clinical-grade drug, α-methyl-L-tyrosine (α-MT), a TH inhibitor, was able to reverse the pathologies in human neurons and TG *Drosophila* models. We also identified opposing effects between LRRK2 and PINK1 on TH expression, suggesting that functional balance between these two genes may regulate the TH–DA pathway.

**Conclusions:**

Our findings highlight the vital role of the TH–DA pathway in PD pathogenesis. LRRK2 and PINK1 have opposing effects on the TH–DA pathway, and its balance affects DA neuron survival. LRRK2 or PINK1 mutations can disrupt this balance, promoting DA neuron demise. Our findings provide support for potential clinical trials using TH–DA pathway inhibitors in early or prodromic PD.

**Supplementary Information:**

The online version contains supplementary material available at 10.1007/s00018-022-04574-x.

## Background

Parkinson’s disease (PD), a common neurodegenerative disease, is characterized by selective and progressive dopamine (DA) neuron loss in the substantia nigra pars compacta (SN) and Lewy body formation [1]. Since the initial description by James Parkinson in 1817, the exact pathophysiologic mechanisms have not been fully elucidated [2]. The key molecular hallmarks include oxidative stress, proteasomal and mitochondrial dysfunction, and protein aggregation [3]. Dysfunction of synaptic vesicle trafficking (SVT) has also been identified [4]. The levels of SVT genes (including PPP2CA, SYNJ1, NSF and PPP3CB) are lower in PD patients’ brains and blood compared to controls [4]. Disturbance in energy metabolism can be predisposing factors [5]. Furthermore, accumulative evidence from studies in patients and experimental models suggests involvement of neuroinflammatory pathways [6–10]. Most PD cases have complex etiologies [11, 12]. Several monogenic forms of PD have been linked to genes including *α-synuclein* (α-syn), *Leucine-rich repeat kinase 2* (LRRK2), *PTEN-induced kinase 1* (PINK1), *Parkin*, *DJ-1*, *Glucosylceramidase beta*, and *Coiled-coil-helix-coiled-coil-helix domain containing 2* [13].

Among the pathogenic genes, *LRRK2* (PARK8) and *PINK1* (PARK6) share some common functions as protein kinases that are involved in multiple signaling pathways. LRRK2 is a large single polypeptide, ubiquitous protein containing multiple domains including ankyrin, leucine-rich and WD40 repeats, a catalytic core Ras-of-complex proteins (ROC) GTPase with serine-threonine kinase activities [14]. The toxicity of LRRK2 mutations is dependent on its kinase activity as shown in various in vitro and in vivo PD models [15]. The increased serine-threonine kinase activity due to a pathogenic G2019S mutation located in the activation loop of LRRK2 kinase domain was first reported in 2005 [16], with more mutations reported to display this toxic gain of function [17]. Pathogenic mutations in ROC-C-terminal of ROC (COR) domains suppress GTP hydrolysis, leading to the protein getting locked in an active state with increased kinase activity [18]. Pharmacological inhibition of LRRK2 kinase activity can alleviate the toxic phenotype resulting from LRRK2 mutations [15]. LRRK2 is involved in multiple signaling pathways via phosphorylation of various substrate proteins including amyloid precursor protein, N-ethylmaleimide sensitive fusion protein, NFATc2, Rb10 Rab5b, Rab7L1, Rab GTPases, leucyl-tRNA synthetase, ribosomal protein S15, Akt1, P53, P62 and tau proteins [19, 20]. However, it is still unclear whether these proteins are bone fide LRRK2 substrates linked to neurodegeneration in PD.

PINK1 was initially recognized as a substrate of phosphatase and tensin homolog deleted on chromosome 10 (PTEN) protein in cancerous cells. The protein is a 68 kDa serine-threonine kinase and mutations of the gene can lead to autosomal recessive, early-onset PD [21]. PINK1 has 581 amino acids containing an N-terminal mitochondria targeting sequence, followed by a transmembrane domain, a serine/threonine kinase domain and regulatory C-terminal domain [22]. Most PD-associated PINK1 mutations are located in its kinase domain, suggesting that its kinase activity is vital to its neuroprotective effects in dopaminergic neurons [22]. PINK1 is involved in maintaining mitochondria homeostasis [23]. PINK1 and Parkin corroborate to promote neuroprotective mitophagy to maintain mitochondria intactness, whereas PINK1 and Parkin mutations impair mitophagy and disturb mitochondria homeostasis, leading to DA neuron vulnerability [24]. The full-length (FL) PINK1 in mitochondria is proteolytically cleaved into a 52 kDa fragment, which is then released into the cytoplasm and subsequently degraded by the proteasome [25]. The extra-mitochondrial PINK1 can regulate tyrosine hydroxylase (TH) expression and DA content in dopaminergic cells in a PINK1 kinase activity-dependent manner [26]. Overexpression of WT PINK1 down-regulated TH expression and decreased DA content in human DA neurons. However, overexpression of PD-related PINK1 mutants increased TH and DA levels, leading to DA neuron vulnerability to stress challenges [26].

Endogenous DA can play pathogenic roles in DA neurodegeneration [27–29]. The DA-dependent neurodegeneration has been validated in PD *Drosophila* models [30]. The endogenous DA promotes α-syn toxicity in DA neurons [28, 31, 32], and also linked to Parkin-induced DA neurodegeneration [33]. DA oxidation induced by overexpression of exogenous tyrosinase impairs dopaminergic cell viability [34]. DA is unstable and can undergo oxidation to produce deleterious oxygen species (ROS) and reactive DA quinones (DAQs), leading to DA neuron vulnerability [27, 35]. DA, which is catalyzed by mitochondrial monoamine oxidase B, can generate reactive and toxic DA metabolite 3,4-dihydroxyphenylacetaldehyde (DOPAL) [36]. DAQs can irreversibly inhibit proteasome activity to impair ubiquitin–proteasome system (UPS) [35, 37] and are involved in iron species-related neurodegeneration [38]. DAQs, DOPAL and ROS react with functional protein residues, leading to protein aggregation, increased oxidative stress and neuronal degeneration [36, 39–41]. Both DAQs and DOPAL covalently conjugate with α-syn to form DA-conjugated α-syn, leading to α-syn oligomerization and formation of toxic α-syn protofibril [42, 43]. Therapeutic agents to inhibit generation and toxicity of DAQs and DOPAL have been shown to be neuroprotective in various PD models [36, 44–46].

In this study, we highlight the important role of the TH–DA pathway in the pathogenesis of PD. LRRK2 and PINK1 have opposing effects on TH expression, and its balance affects DA neuron survival. LRRK2 and PINK1 mutations disrupt the balance and promote neurodegeneration, which can be rescued by TH inhibitor α-methyl-tyrosine (α-MT) and WT PINK1.

## Materials and methods

### Plasmids and vectors

To generate human LRRK2 and PINK1 stable transfected human dopaminergic SH-SY5Y cell lines, wild-type (WT) FL human LRRK2 and PINK1 cDNA was cloned into pcDNA3.1(-) mammalian expression vectors (Invitrogen) using In-Fusion cloning protocol (TaKaRa Bio Inc) and G2019S LRRK2 mutation, and A339T and E231G PINK1 mutations were created by polymerase chain reaction (PCR)-based technique using QuikChange® site-directed mutagenesis kit (Stratagen) as previously reported [26]. For transient transfection of PC12 cells, 2XMyc-LRRK2-WT (Addgene plasmid 25361), 2XMyc-LRRK2-G2019S (Addgene plasmid 25362), 2XMyc-LRRK2-3XKD (Addgene plasmid 25366), 2XMyc-LRRK2-kinase (Addgene plasmid 25071) and 2XMyc-LRRK2-RCK (Addgene plasmid 25065) plasmids developed by Dr. Mark Cookson were purchased from Addgene. 2XMyc-LRRK2-Kinase-G2019S, 2XMyc-LRRK2-Kinase-3XKD, 2XMyc-LRRK2-RCK-G2019S and 2XMyc-LRRK2-RCK-3XKD vectors were created using 2XMyc-LRRK2-kinase (Addgene plasmid 25071) and 2XMyc-LRRK2-RCK (Addgene plasmid 25065) as templates, respectively, based on QuikChange® site-directed mutagenesis protocol (Stratagen). Primers were designed and manipulated by Primer Premier 5.0, SimVector 4 and DNAMAN softwares. Primers used for plasmids cloning, site-directed mutagenesis and sequencing validation experiments are shown in Supplementary Table 1, Supplementary Table 2 and Supplementary Table 3. All positive clone sequencing were verified before performance of transfection protocols.

### LRRK2 and PINK1 SH-SY5Y stable cell lines

The protocol to create human dopaminergic SH-SY5Y LRRK2 and PINK1 stable cell lines used in the current study had been reported previously [26]. Briefly, SH-SY5Y cells were transfected with pcDNA3.1 vector, pcDNA3.1-LRRK2-WT, pcDNA3.1-LRRK2-G2019S, pcDNA3.1-PINK1-WT, pcDNA3.1-PINK1-A339T or pcDNA3.1-PINK1-E231G vectors, respectively, using Lipofectamine reagents. After transfection, cells were treated with selection medium containing 0.6 g/L (w/v) G418 antibiotics (Promega) for 2–3 weeks before resistant cells were separated by serial dilution and allowed to grow from single cells. The stably transformed clones were verified by real time RT-PCR and Western blot analysis. Cells were maintained at 37 °C in a 5% CO_2_ incubator with 0.4 g/L (w/v) G418.

### Drug administrations

To challenge cells with H_2_O_2_ or iron species, cells were treated with 100 µM H_2_O_2_, 100 µM FeSO_4_ or 100 µM FeCl_3_, respectively, and incubated for 24 h at 37 °C before analysis of cell viability or rate of cell death. To deplete DA in dopaminergic SH-SY5Y cells, cells were maintained in Dulbecco's modified Eagle medium (DMEM) in the presence of 1 mM α-MT for several passages before being used for experiments. To modulate DA content in *Drosophila* heads, *Drosophila* were cultured in culture mediums with or without 15 µM, 500 µM, 2 or 4 mM α-MT for different time periods before withdrawal of α-MT or checking of *Drosophila* PD symptoms and monitoring DA content in *Drosophila* heads. To study the reciprocal regulations of PINK1 and LRRK2 protein levels via the proteasome pathway, LRRK2 or PINK1 stable SH-SY5Y cells were cultured in the presence or absence of 3 µM MG132 for 6 h before analysis of LRRK2 or PINK1 protein levels by Western blot analysis. All chemicals were purchased from Sigma-Aldrich.

### Reprogramming of induced pluripotent stem cells (iPSC)

The collection of human peripheral blood samples followed the inclusion criteria as per the SingHealth CIRB Ref NO: 2018/2920. Briefly, 2 ml of peripheral blood of patients with or without G2019S LRRK2 mutation was collected and lysed in 2 ml of 1 × red blood cell lysis buffer (eBioscience, San Diego, CA) for 10 min. After lysis, peripheral blood mononuclear cells were spun down and harvested. About 30,000 cells with or without G2019S LRRK2 mutation were suspended in 500 µl of StemSpan expansion medium (StemCell Technologies, Vancouver) in 1 well of a 24-well tissue culture plate and infected with OCT4, SOX2, KLF4 and cMYC Sendai virus (CytoTune-iPS Reprogramming Kit, Thermo Fisher Scientific) with a multiplicity of infection of ten. 24 h later, the infected cells were cultured in 0.5 ml fresh cell expansion medium and plated onto Matrigel (BD Biosciences)-coated dish 2 days later. The induced pluripotent stem cell colonies with an embryonic stem cell (ESC)-like appearance were identified and isolated manually between day 18 to day 25 post-infection. The iPSCs were maintained on Matrigel-coated plates in mTESR-1 medium (Stem Cell Technologies). Cells were maintained at 37 °C in a humidified atmosphere containing 5% CO_2_.

### Neural progenitor cell (NPC) induction and dopaminergic neuron differentiation

For NPC induction, iPSCs with or without G2019S LRRK2 mutation were harvested using a cell scraper and cultured in suspension as embryoid bodies (EBs) for 8 days in StemPro defined medium (Thermo fisher scientific) minus fibroblast growth factor 2 (FGF2). EBs will then be cultured for an additional 2–3 days in suspension in neural induction medium containing DMEM/F12 with Glutamax, NEAA, N2 and 20 ng/ml FGF2 before attachment on Matrigel-coated culture plates. Neural rosettes were isolated manually with glass Pasteur pipette 3 days later and dissociated into single cells under Accutase digestion and replated onto culture dishes to obtain a homogeneous population of NPC. The NPCs were expanded in Neurobasal media containing nonessential amino acids (NEAA), 2 mM glutamine, B27 and 20 ng/ml FGF2. To induce dopaminergic differentiation, NPCs were plated onto poly-D-lysine and mouse laminin-coated dishes and cultured in DA medium I: Neurobasal medium supplemented with 2 mM NEAA, l-glutamine, 2% B27, 200 ng/ml sonic hedgehog (SHH) and 100 ng/ml FGF8 for 7 days. After 7 days, the DA progenitor cells were then fed with DA medium II: neurobasal medium supplemented with 2 mM NEAA, L-glutamine, 2% B27 and brain-derived neurotrophic factor (BDNF) and glial cell-derived neurotrophic factor (GDNF) (20 ng/ml of each), 1 ng/ml transforming growth factor beta-3, 200 µM ascorbic acid and 1 mM cAMP for 6 weeks before Western blot analysis or high-performance liquid chromatography (HPLC) analysis of DA content in DA neurons.

### Generation of human middle brain-like organoids (hMLOs)

hMLOs were generated from human pluripotent stem cells using a previously reported protocol with minor modifications [47]. Briefly, hPSCs were dissociated from intact colonies to single cells using TrypLETM Express (Gibco), and 10,000 cells were plated in each well of a low-cell-adhesion 96-well culture plate with V-bottomed conical wells (Sumitomo Bakelite) to form uniform embryoid bodies (EBs) in a medium containing DMEM/F12:Neurobasal (1: 1), 1:100 N2 (Invitrogen), 1:50 B27 without vitamin A (Invitrogen), 1% GlutaMAX (Invitrogen), 1% minimum essential media–nonessential amino acid (Invitrogen) and 0.1% β-mercaptoethanol (Invitrogen) supplemented with 1 μg/ml heparin (Sigma-Aldrich), 10 μM SB431542 (Stemgent), 200 ng/ml Noggin (R&D Systems), 0.8 μM CHIR99021 (STEMCELL Technologies) and 10 μM ROCK inhibitor thiazovivin (Tocris). On day 4, midbrain patterning media that contain additional components (100 ng/ml SHH-C25II (R&D Systems), 100 ng/ml fibroblast growth factor 8 (FGF8) (R&D Systems) and 0.5 μM purmorphamine (Stemgent)) were added. After 3 days, the resulting neurospheroids were embedded in 30 μl of reduced growth factor Matrigel and placed in a 37 °C incubator for 30 min to solidify Matrigel, and the tissue growth induction media containing Neurobasal medium, 1:100 N2 supplement, 1: 50 B27 without vitamin A, 1% GlutaMAX, 1% minimum essential media–nonessential amino acid, and 0.1% β-mercaptoethanol supplemented with 2.5 μg/ml insulin (Sigma-Aldrich), 200 ng/ml laminin (Sigma-Aldrich), 100 ng/ml SHH-C25II, 100 ng/ml FGF8 and 0.5 μM purmorphamine were added. After 24 h, the hMLOs were transferred into ultralow-attachment six-well-plates (Costar) containing the final differentiation media, which consisted of Neurobasal medium, 1:100 N2 supplement, 1: 50 B27 without vitamin A, 1% GlutaMAX, 1% minimum essential media–nonessential amino acid, 100 U/ml penicillin G and 100 μg/ml streptomycin, and 0.1% β-mercaptoethanol with supplements [10 ng/ml BDNF (Peprotech), 10 ng/ml GDNF (Peprotech), 100 μM ascorbic acid (Sigma-Aldrich) and 125 μM db-cAMP (Sigma-Aldrich)], and cultured on an orbital shaker. From day 30, the amount of N2 and B27 without vitamin A was reduced into half, and the hMLOs were maintained without supplements from day 60 onward. The medium was replaced every 3 days.

### Transgenic (TG) LRRK2 mice lines

LRRK2 G2019S TG mice were generated using a bacterial artificial chromosome containing the entire mouse mutant G2019S LRRK2 and were purchased from the Jackson Laboratory (#012467) [48]. LRRK2 R1441G TG mice generated from BAC containing the entire human mutant R1441G LRRK2 were also provided by the Jackson Laboratory (#009604) [49]. Mice genotypes were verified by PCR using genomic DNA from mice tails. Mice were maintained in a pathogen-free facility and exposed to a 12 h light/dark cycle with food and water. All procedures were performed in accordance with institutional guidelines, and all protocols were approved by the Institutional Animal Care and Use Committee (IACUC) of the National Neuroscience Institute (NNI) of Tan Tock Seng Hospital (TTSH).

### *Drosophila* stocks, preparation and behavioral assays

Promoter lines containing elav-GAL4, ddc-GAL4 and 24B-GAL4 were obtained from Bloomington Stock Center (Bloomington, IN, USA). Transgenic human LRRK2 *Drosophila* were generated by *Drosophila* embryo microinjection protocols as previously described [50]. Briefly, WT or mutant G2019S LRRK2 human cDNA containing a myc-tag at the C terminus was cloned into pUAST plasmid and microinjected into *Drosophila* embryos (BestGene. USA). Positive lines were selected and sequence confirmed before culture for experiments. The UAS-HA-PINK1/cyo *Drosophila* line to express *Drosophila* PINK1 was a gift from Prof Alexander J Whitworth (MRC Centre for Developmental and Biomedical Genetics, Sheffield S10 2TN, UK). The UAS-hPINK1 309D7C1 II *Drosophila* line to overexpress mutant G309D PINK1 was a gift from Prof Zhuo hua Zhang (Centre for Medical Genetics and Hunan Key Laboratory of Medical Genetics, School of Life Sciences, Central South University, Changsha, Hunan 410008, China). The UAS-hPINK1 and dPINK1 RNAi (II, III) *Drosophila* lines were gifts from Prof Lu Bing Wei (Stanford University. USA). To create the LRRK2 plus PINK1 double transgenic *Drosophila* lines, stable LRRK2 and PINK1 1 *Drosophila* were first achieved, respectively, via the following protocol. The promoter ddc-GAL4 line was crossed with the UAS controlled LRRK2 and PINK1 lines, respectively. The red eye virgin offspring *Drosophila* were selected and crossed with TM3/TM6B balancer males. Then progeny males with the reddest eye color were single picked and crossed with balancer lyshid/TM6B virgins in individual vials. On day 6, the individual vials were heat shocked at 37 ºC to achieve pure stable gene stocks. Stable lines were verified using single *Drosophila* PCR and Western blot experiments. The stable LRRK2 and stable PINK1 lines were then crossed to obtain the double LRRK2/PINK1 *Drosophila* lines. *Drosophila* were routinely raised at 25 °C on cornmeal media that were replaced every 3 days. *Drosophila* heads were harvested at the respective time points for HPLC analysis of DA content, confocal microscopy detection of TH-positive DA neurons and Western blot analysis of TH, PINK1 or LRRK2 proteins in *Drosophila* heads. For longevity experiments, 1 day-old *Drosophila* were transferred to fresh media every 3 days. The life span was assessed by monitoring the survival of 50 *Drosophila* in five cohorts for each genotype. The triplicate cohorts of 50 *Drosophila* per genotype were monitored for survival daily. For the climbing assay, motor ability was assessed at 15 min intervals using the negative geotaxis assay. Briefly, three cohorts of 20 female and age-matched control *Drosophila* were anesthetized and placed in a vertical plastic column (length, 25 cm; diameter, 1.5 cm). After a 2 h recovery period, *Drosophila* were tapped to the bottom and the percentage of *Drosophila* that climbed to or above the top column line in 1 min was calculated. Triplicate trials were performed in each experiment at 15 min intervals.

### Immunochemistry staining of TH-positive DA neurons in *Drosophila* heads

*Drosophila* brain fixation and antibody staining were carried out as described previously [51]. Briefly, adult brains were dissected in phosphate-buffered saline (PBS), then fixed with 4% formaldehyde and washed a few times with PBT (PBS + 0.1% Triton X-100). After blocking with 3% BSA (in PBT), *Drosophila* brain samples were incubated in primary antibody (rabbit anti-TH; Sigma-Aldrich; T8700, 1: 1000 dilution) overnight at 4 °C with rotation, washed with PBT and incubated in secondary antibody (Cy3-conjugated goat anti-rabbit; Jackson Immunoresearch; 1:500). Samples were washed a few times with PBT, incubated with Vectashield and TH-positive DA neurons were analyzed by Carl Zeiss Upright Confocal Microscope (Zeiss).

### Analysis of insoluble ROS-modified and DAQ-conjugated proteins in *Drosophila* heads by Western blot analysis

*Drosophila* heads were homogenized and lysed in buffer (25 mM Tris–HCl, pH 7.4, 150 mM NaCl, 0.1% (w/v) sodium dodecyl sulfate (SDS)) containing protease inhibitors (Roche Applied Science) and phenylmethylsulfonyl fluoride and incubated on ice for 30 min. The lysate was then sonicated before centrifugation at 13,200 rpm (16,000* g*) for 30 min to obtain the soluble (supernatant) and insoluble (pellet) fractions. The pellet was solubilized in 30 µl of 4 × SDS loading buffer (161–0737, BIO-RAD) with continuous shaking at 4 °C overnight, then boiled and loaded for gel electrophoresis, SDS-PAGE and Western blot analysis with anti-DA antibody (Abcam, ab8888, 1: 1000–2000) to detect DA-conjugated proteins or with anti-dinitrophenylhydrazone (DNP) antibody (Cell biolabs, 230801, 1:1000) to detect ROS-modified carbonylated proteins, respectively.

### Western blot analysis

Cells, *Drosophila* heads, brain organoids or mice brain tissues were collected in lysis buffer (100 mM HEPES pH 7.5, 5 mMmagnesium chloride, 150 mMsodium chloride, 1 mMEDTA, 1% (v/v) Triton X-100 and 1% (v/v) protease inhibitor cocktail (Calbiochem)) and centrifuged at 12,000* g* at 4 °C for 10 min. From the supernatant, 30 μg proteins was resolved by 15% SDS-PAGE. The proteins were transferred onto a nitrocellulose membrane using an electric blotting apparatus (Biorad), blocked with washing buffer A (150 mM sodium chloride plus 13 mMTris–hydrochloric acid, pH 7.5, and 0.1% (v/v) Tween 20) containing 5% (w/v) skim milk for 30 min (for TH, GFP and PINK1) or 1 h (for caspase-3) at room temperature before incubation with primary antibody in washing buffer A with 2% (w/v) skim milk (rabbit-anti-PINK1 antibody, Novas Biologicals. 1:1000; or anti-TH (for *Drosophila* samples), Sigma-Aldrich, T8700, 1:1000; anti-TH (for brain organoids, mouse brain and DA cell samples), Santa Cruz Biotechnology, sc-25269, 1:1000; anti-LRRK2 (for mice brain samples), Abcam, ab133474, 1:10,000, anti-LRRK2 (for cells and *Drosophila* samples), Sigma-Aldrich, HPA014293, 1:1000) overnight at 4 °C. The membrane was washed 5 × 5 min each with washing buffer A and subsequently incubated with the secondary antibody (anti-mouse antibody, Santa Cruz Biotechnology; sc-2005, 1:5000 or anti-rabbit antibody, Sigma-Aldrich, A4914, 1: 10,000) for 2 h at room temperature. Following subsequent washes, the blots were developed with the enhanced chemiluminescent kit (Pierce) on Kodak CL-Xposure^™^ films.

### Quantitative densitometric analysis of Western blot data

Quantitative analysis of the densities of protein bands in Western blot gels was done by densitometric analysis using the Image software Bandscan 4.30. The relative densities of the respective protein bands of LRRK2, PINK1 and TH in control lanes in Western blot gels were set as 50%. The relative densities of protein bands of LRRK2, PINK1 and TH of other lanes in Western blot gels were expressed as the ratio of the densities for LRRK2, PINK1 and TH, after automatic comparison with the ratio of densities of control lanes by the software.

### Quantitative real-time reverse transcription PCR (RT-PCR)

Expression levels of LRRK2 and TH in SH-SY5Y and PC12 cells were analyzed using real-time quantitative RT-PCR. Total RNA was isolated using NucleoSpin RNA II (Macherey–Nagel). A two-step quantitative RT-PCR was carried out. Reverse transcription was performed with Maxima First-Strand cDNA synthesis kit for RT-PCR (Fermentas); 1 µg RNA sample was used for each reverse transcription. RT-PCR primers used in this study were acquired from PrimerBank (http://pga.mgh.harvard.edu/primerbank/) as shown in Supplementary Table 4. All real-time PCR reactions were performed using the CFX96 Real-Time PCR detection system (Bio-Rad Laboratories) and the amplifications were done using Maxima SYBR Green/Fluorescein qPCR master mix (2X) (Fermentas). The thermal cycling conditions were composed of 50 ºC for 2 min, followed by an initial denaturation step at 95 ºC for 10 min, 40 cycles at 95 ºC for 15 s and 60 ºC for 45 s. The experiments were carried out in triplicate for each data point. Expression data were normalized to the geometric mean of housekeeping gene β-actin to control the variability in expression levels. The relative quantification in gene expression was determined using the 2^−ΔΔCT^ method [52]. The expression levels of TH or LRRK2 of cells transfected with expression or shRNA vectors of PINK1 were expressed as % of control cells, set as 100%.

### HPLC analysis of DA contents

The HPLC analysis was performed using SHIMADZU prominence LC-20 HPLC system (SHIMADZU) equipped with an Esa Coulochem 5300 electrochemical detector and a reversed-phase column (70–0636 MD-150 3.2 × 150 mm 3 µm, Dionex) and analyzed under the control of LC Solution program. The setting of ECD was as follows: guard cell: Model 5020, analytical cell: Model 5014B, potentials: E1 = − 300 mV, E2 =  + 200 mV, *E*_GC_ =  + 350 mV, and analyzing range was set as 5 uA. The SHIMADZU HPLC system was formed by LC-20AD Liquid Chromatograph, SIL-20AC Prominence Autosampler, CTO-20AC Prominence Column Oven, CBM-20A Prominence Communication Bus Module and DGU-20A_5_ Prominence Degasser. During the HPLC procedure, isocratic elution with elution buffer (100 mM sodium phosphate, 10 mM sodium hepatosulfate, 0.1 mM EDTA, adjusted by phosphoric acid to pH 2.95, 8% (v/v) methanol) was performed. All solutions for HPLC analysis were filtered through GNWP04700 NYLON 20UM WH PL 47MM membrane (Millipore) before use. Cells, *Drosophila* heads and mice brain or human brain organoid samples were harvested, homogenized in 100 µl ice-cold 0.5 N perchloric acid (Roche), sonicated (Vibra-Cell VCX130, Sonics & Materials) for 1 min in ice and centrifuged; 40 μl of filtered lysate was loaded and analyzed under a flow rate of 0.5 ml per min for 15 min for each sample. The DA peak appeared in the HPLC chromatograph at about 13 min of elution time. The DA content in the solutions was acquired based on the DA peak areas in HPLC chromatography. DA content in cells transfected with empty vectors or pcDNA3-EGFP only acted as control groups. The DA peak areas of control groups were set as 100%, while the DA peak areas of other groups were calculated and expressed as % of control groups.

### MTT assay

MTT (3-(4,5-dimethylthiazol-2-yl)-2,5-diphenyltetrazolium bromide, Sigma) was prepared as 2 mg/ml stock solution in PBS and stored at 4ºC. The MTT/DMEM solution (15% (v/v) of 2 mg/ml MTT stock solution mixed with 85% (v/v) DMEM) was prepared freshly. Cells in 24-well dishes were washed with PBS and incubated in 500 µl of MTT/DMEM solution in the dark at 37 ºC for 3 h. The solution was then aspirated without disrupting the cells and 500 µl of solubilizing solution (0.04 M hydrochloric acid/isopropanol plus 3% (w/v) SDS) was added and mixed well. The plates were incubated at room temperature for 1 h in the dark. The solution optical density was measured at 595 nm using a spectrophotometer (Elisa Reader Spectra Max 340, U.S.A.) in a 96-well plate (Iwaki). The cell viability of control groups was set as 100%, while the cell viability of other groups was set as a percentage of that of control cells.

### Trypan blue exclusion analysis

Cells were harvested by trypsinization. The cell suspension was mixed with the same volume of 2% w/v trypan blue solution and cells were counted under a light microscope (Axiovert 25 Zeiss). The numbers of total cells and dead cells were counted, respectively, and the percentage of dead cells was determined. At least, 200 cells were counted for each cell suspension sample. At least six replicates were used per sample.

### Statistical analysis

Statistical analyses were conducted using one-way or two-way ANOVA, followed by post hoc Dunnett’s test using software Minitab 14. Graphs were constructed with SigmaPlot 2001.

## Results

### LRRK2 G2019S mutation up-regulated TH and DA and promoted neuronal death in cell models

**Fig. 1:**
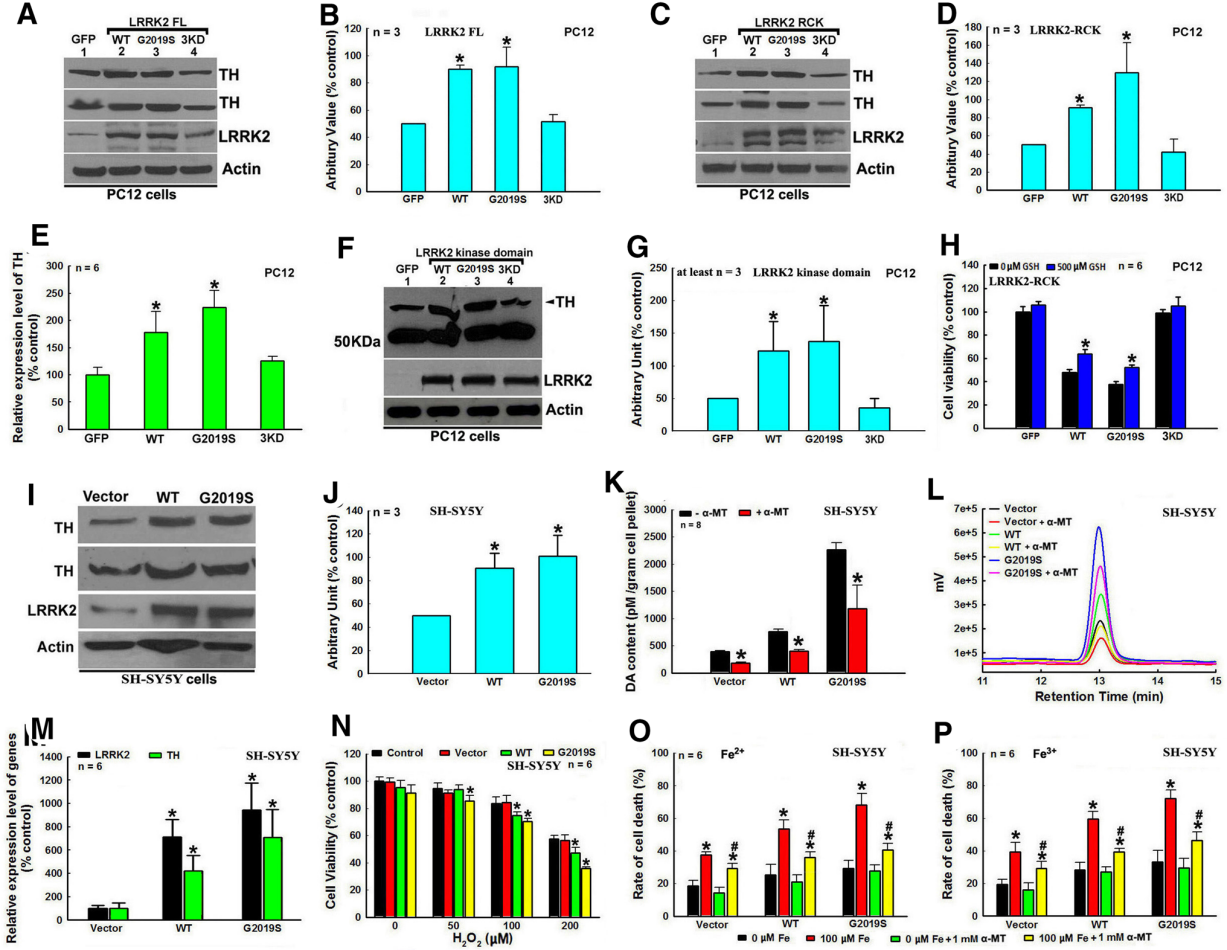
LRRK2 G2019S mutation deregulates the TH–DA pathway, leading to DA neuron vulnerability. **A**–**G** LRRK2 enhances TH expression in PC12 cells in an LRRK2 kinase activity-dependent manner. **A**, **C** and **F** Representative Western blot data of up-regulated TH levels in PC12 cells under transient overexpression of WT, mutant G2019S or 3KD LRRK2. **A** FL LRRK2, **C** LRRK2 RCK domains and **F** LRRK2 kinase domain. **B**, **D** and **G**, Densitometric analysis of TH protein bands of Western blot gels in **A**, **C** and **F**, respectively. *, at least *P* < 0.01, compared with arbitrary value of TH protein bands of GFP-transfected control cells. **E** Quantitative real-time RT-PCR analysis of TH expression in PC12 cells under overexpression of WT, mutant G2019S or 3KD LRRK2 RCK domains. *P < 0.01, compared with relative TH expression level of GFP-transfected control cells. **H** Expression of WT and mutant G2019S LRRK2 RCK domains impair PC12 cell viability, which can be partially rescued by 500 µM GSH treatment. *, at least *P* < 0.01, compared with cell viability of the respective WT or G2019S RCK domains transfected PC12 cells in the absence of GSH treatment. (I–P) Stable transfection of LRRK2 up-regulates TH and DA levels, sensitizing human dopaminergic SH-SY5Y cells to stress challenges. **I** Representative Western blot TH protein bands in SH-SY5Y cells stably transfected with empty vector, WT or mutant G2019S FL LRRK2. **J** Densitometric analysis of Western blot TH protein bands in **I**. *P < 0.001, compared with arbitrary value of TH bands of empty vector transfected control SH-SY5Y cells. **K** and **L** Mutant G2019S LRRK2-induced DA content increase in stably transfected SH-SY5Y cells can be abrogated by treatment with 2 mM α-MT, a specific TH inhibitor. **K** Quantitative analysis of DA content by HPLC, *, at least *P* < 0.05, compared with DA content of the respective cells in the absence of α-MT. **L** Representative HPLC chromatography of DA peaks. **M** Quantitative real-time RT-PCR analysis of TH and LRRK2 expression level in stably transfected SH-SY5Y cells. *, at least *P* < 0.01, compared with TH and LRRK2 expression levels of empty vector transfected SH-SY5Y cells. **N** Stable transfection of mutant G2019S LRRK2 sensitizes SH-SY5Y cells to H_2_O_2_ challenge. *, at least *P* < 0.05, compared with cell viability of empty vector transfected SH-SY5Y cells treated with the respective dosage of H_2_O_2_. (**O** and **P**) 2 mM α-MT treatment alleviates LRRK2-induced SH-SY5Y cell viability impairment under 100 µM Fe^2+^ (**O**) or 100 µM Fe^3+^ (**P**) challenges. *, at least *P* < 0.05, compared with cell death rate of the respective stable cells without iron species challenges. #, at least *P* < 0.05, compared with cell death rate of respective SH-SY5Y cells under the respective iron species challenges

We showed that LRRK2 up-regulated TH expression and DA in dopaminergic neurons in a kinase-dependent manner and promoted neuronal degeneration (Fig. 1). The transient overexpression of FL, Rock-Cor-kinase (RCK) LRRK2 domains or only kinase domain of human WT or mutant G2019S LRRK2, but not 3 kinase dead (KD) LRRK2, increased TH expression in dopaminergic PC12 cells and impaired cell viability, which can be partially rescued by GSH treatment (Fig. 1A–H). Furthermore, stable overexpression of FL WT or mutant G2019S human LRRK2, especially G2019S mutant LRRK2, increased TH expression and DA in human dopaminergic SH-SH5Y cells, sensitizing cells to H_2_O_2_- and iron species-induced stress challenges (Fig. 1I–P). However, LRRK2 up-regulated DA and the resulting increased vulnerability of SH-SY5Y cells can be abrogated by treatment with 1 mM α-MT, a specific TH inhibitor (Fig. 1K, L, O and P). We also demonstrated that mutant G2019S LRRK2-induced up-regulation of TH expression cannot be reversed by GSH or α-MT treatments (Supplementary Fig. 1).

### LRRK2 G2019S mutation promoted DA neurodegeneration via the TH–DA pathway in *Drosophila* PD model

**Fig. 2:**
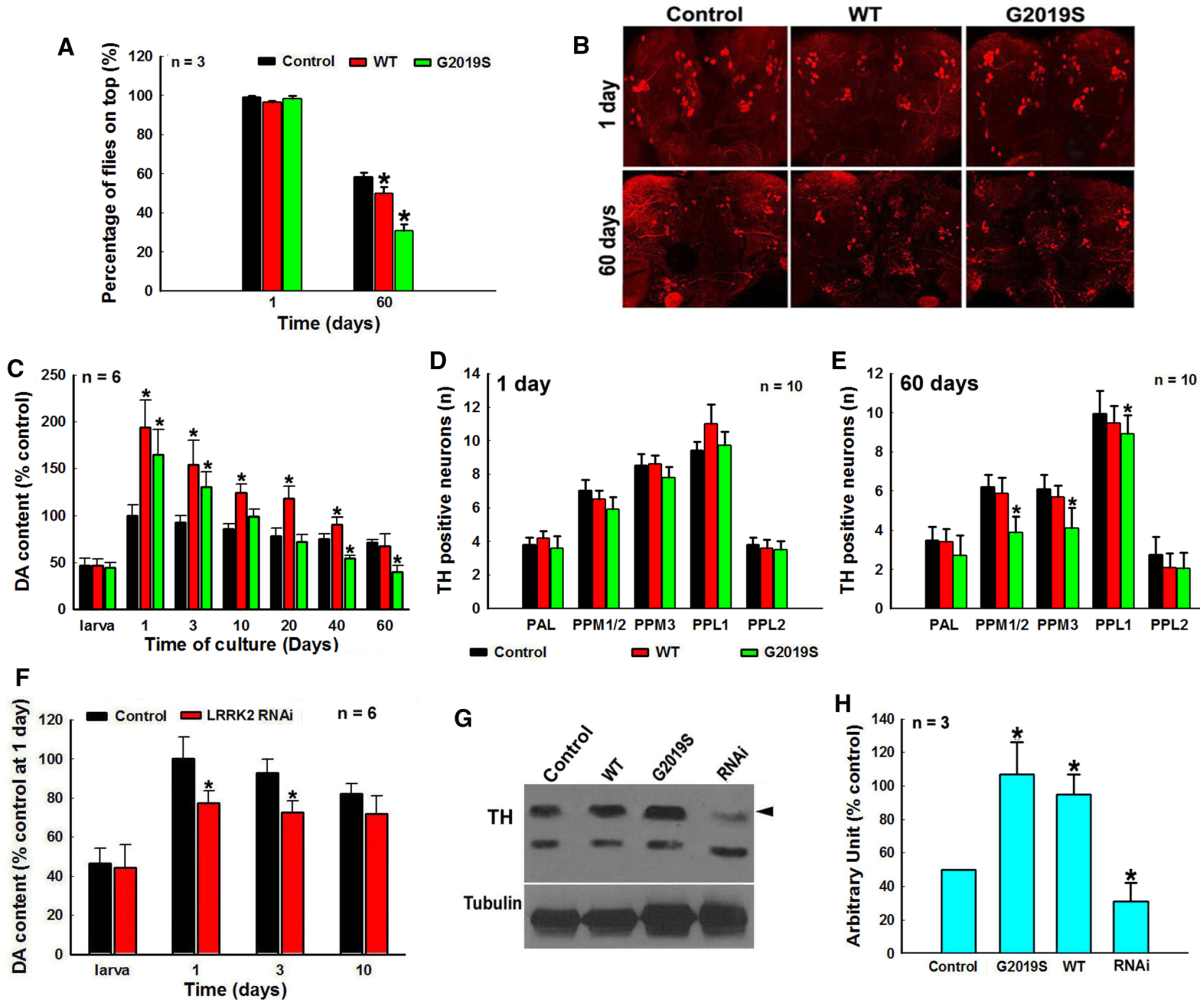
LRRK2 G2019S mutation deregulates the TH–DA pathway, contributing to DA neurodegeneration in *Drosophila* PD model. Yellow white control, LRRK2 TG *Drosophila* and LRRK2 RNAi *Drosophila* lines are crossed with ddc-GAL4 line to modulate LRRK2 expression in *Drosophila* head DA neurons and cultured for different time periods. **A** Locomotor deficits of climbing ability of *Drosophila* with or without TG expression of human WT and G2019S LRRK2 in *Drosophila* head DA neurons after 1 day and 60 days culture. *, at least *P* < 0.05, compared with the percentage of *Drosophila* on top of the control group. **B** Representative confocal image of TH neurons in *Drosophila* heads of control and LRRK2 TG *Drosophila* cultured for 1 day or 60 days. **C** HPLC analysis of DA content in *Drosophila* heads of control and LRRK2 TG *Drosophila* at different culture time points. *, at least *P* < 0.05, compared with DA content in *Drosophila* heads of control group at the respective time points. **D** and **E** Number of TH-positive dopaminergic neurons in *Drosophila* heads cultured for 1 day (**D**) and 60 days (**E**), *, at least *P* < 0.05, compared with the number of TH positive neurons in control *Drosophila* heads cultured for 60 days. **F** HPLC analysis of DA content in *Drosophila* heads of control and LRRK2 RNAi *Drosophila* lines. *P < 0.05, compared with DA content in *Drosophila* heads of control group at the respective culture time points. **G** Representative Western blot TH levels in *Drosophila* heads of 3 days cultured control, TG LRRK2 and LRRK2 RNAi *Drosophila* lines. **H** Densitometric analysis of TH bands in Western blot gels in G. *, at least *P* < 0.05, compared with the arbitrary value of TH bands in control *Drosophila* heads

**Fig. 3:**
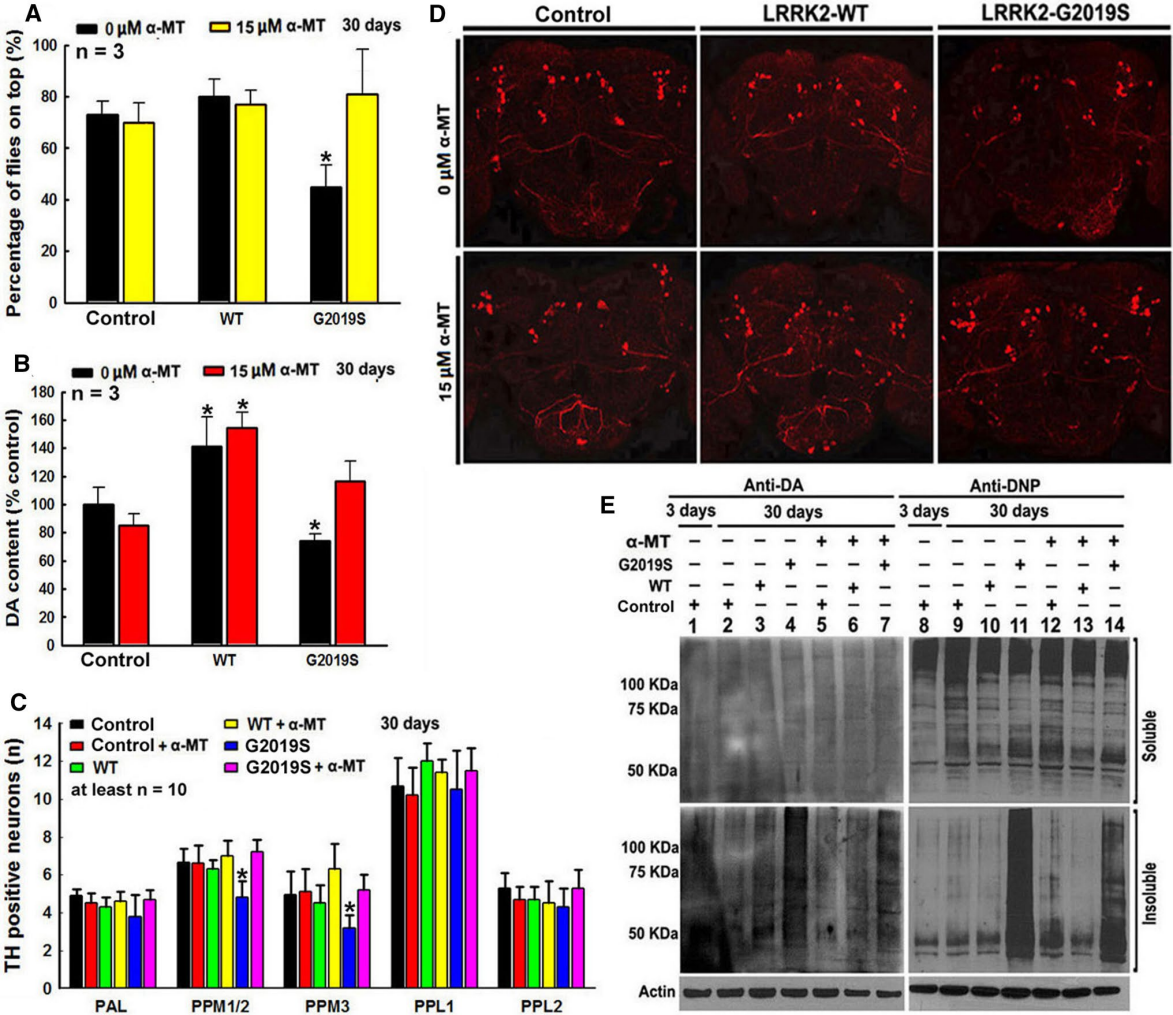
LRRK2 G2019S mutation-induced *Drosophila* DA neurodegeneration can be abrogated by α-MT, a TH inhibitor. Yellow white control and WT or mutant G2019S LRRK2 TG *Drosophila* are crossed with ddc-GAL4 line and cultured in the presence or absence of 15 µM α-MT for 30 days. **A** α-MT treatment can improve locomotor deficits of climbing ability of TG mutant G2019S LRRK2 *Drosophila*. *P < 0.01, compared with the percentage of *Drosophila* on top of control group in the absence of α-MT. **B** α-MT treatment can prevent mutant G2019S LRRK2-induced DA content decrease in *Drosophila* heads. *, at least *P* < 0.05, compared with DA content in *Drosophila* heads of control group in the absence ofα-MT. **C** α-MT treatment can protect against DA neurodegeneration in mutant G2019S LRRK2 *Drosophila* heads. *, at least *P* < 0.05, compared with the number of TH-positive neurons in control *Drosophila* heads. **D** Representative confocal image of TH neurons in *Drosophila* heads of control and LRRK2 transgenic *Drosophila* cultured for 60 days. **E** Representative Western blot gels of DA-conjugated proteins and DNP-positive proteins in control and LRRK2 TG *Drosophila* heads after culture for 3 days or 30 days

Next, we studied how LRRK2 mutation affected deregulation of the TH–DA pathway and DA neurodegeneration in transgenic (TG) PD *Drosophila* models (Fig. 2 and 3). Overexpression of human WT or mutant G2019S LRRK2 in *Drosophila* DA neurons for 60 days induced PD-like phenotype and DA neuron loss (Fig. 2A–E). Overexpression of G2019S mutant up-regulated TH protein level and DA at early stage (Fig. 2C and G). However the DA content in G2019S mutant subsequently decreased rapidly over time (Fig. 2C). At 60 days culture, DA in G2019S mutant was lower than that of controls, accompanied by the loss of DA neurons (Fig. 2B, C and E). RNAi knockdown (KD) of LRRK2 decreased DA and TH levels without significant effect on DA neuron development (Fig. 2F and G, Supplementary Fig. 2).

### LRRK2 G2019S mutation-induced *Drosophila* DA neurodegeneration can be abrogated by α-MT, a TH inhibitor

Subsequently, TH inhibitor α-MT was used to determine if deregulation of the TH–DA pathway was responsible for LRRK2 mutation-induced DA cell death (Fig. 3). α-MT is an orally active competitive TH inhibitor, which has been used to control hypertensive symptoms in pheochromocytoma patients [53]. The α-MT-induced movement side effects (such as tremor) can be reversed after stopping the drug or with high dosage L-tyrosine [54]. In our study, treatment of *Drosophila* with higher dosage α-MT (500 µM, 2 mM and 4 mM) led to a dosage-dependent decline of their climbing capability and a decrease in DA content in *Drosophila* heads (Supplementary Fig. 3). However, after α-MT withdrawal, their climbing capability and DA normalized (Supplementary Fig. 3), suggesting that α-MT can reversibly inhibit TH (Supplementary Fig. 3). After dosage optimization, 15 µM low dosage α-MT was selected and we showed that this dosage significantly protected against mutant G2019S-induced DA degeneration (DA neuron loss, PD phenotype and late-stage decrease in DA content) (Fig. 3A–D). Low-dose α-MT did not affect the life span of NTG control *Drosophila*, but increased the life span of G2019S *Drosophila* (Supplementary Fig. 4). Previous studies had shown that increased DA production can generate reactive DA metabolites, contributing to DA toxicity [35]. We found insoluble DA-conjugated and ROS-modified proteins in G2019S *Drosophila* after 30 days (Fig. 3E), and these were reversed by low-dose α-MT (Fig. 3E).

### LRRK2 mutations dysregulate the TH–DA pathway in TG mice models

**Fig. 4:**
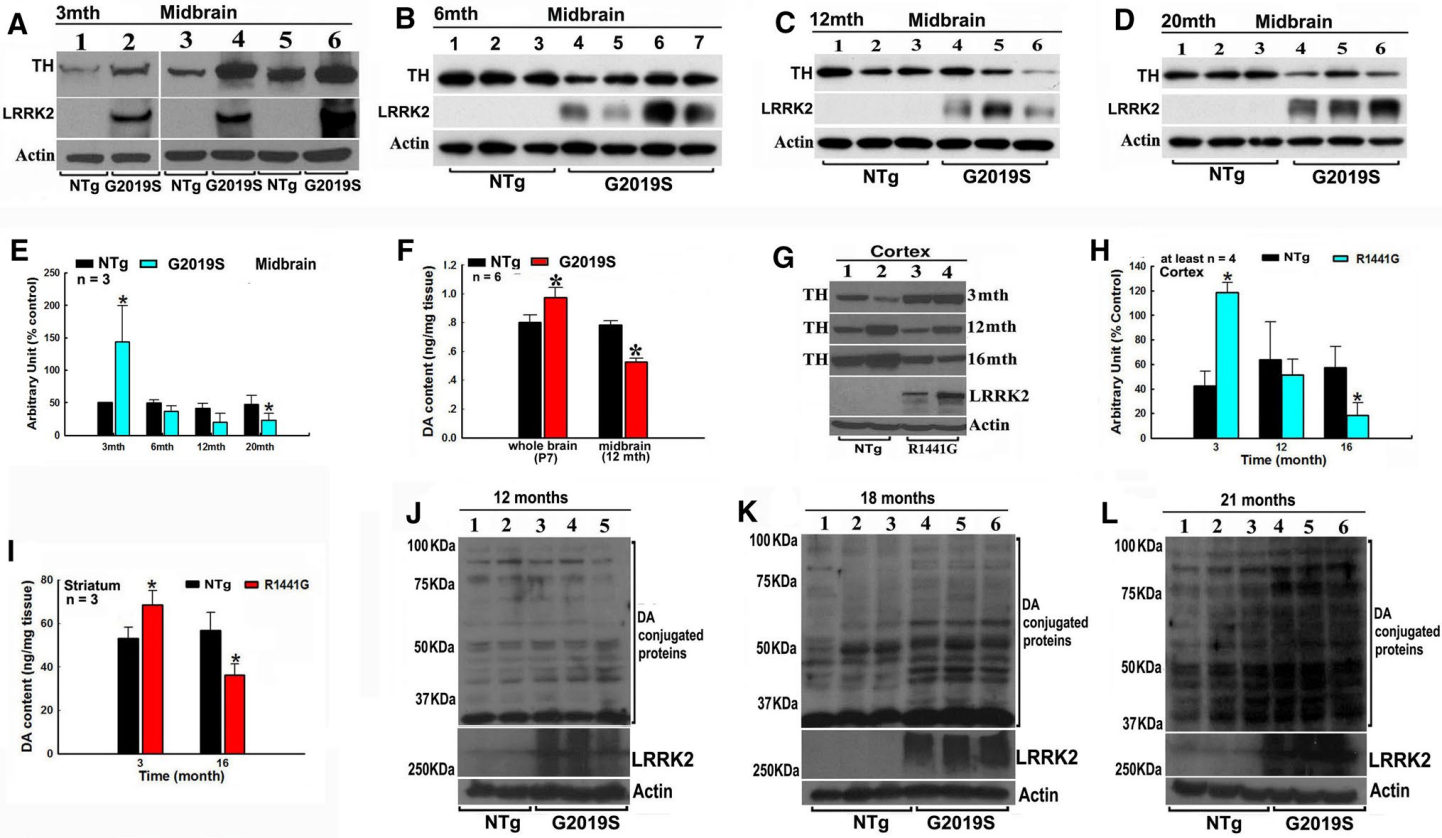
PD-linked LRRK2 mutations-induced deregulation of the TH–DA pathway in TG mice models. **A**–**F** G2019S LRRK2 mutation induces age-relevant alterations of TH and DA levels in transgenic mice midbrains. **A**–**D** Western blot data of TH protein levels in TG mice midbrains. **A** 3 months (3 mth), **B** 6 months (6mth), **C** 12 months (12 mth), **D** 20 months (20 mth). **E** Densitometric analysis of TH protein bands in Western blot gels in A–E. *, at least *P* < 0.05, compared with arbitrary value of TH bands of non-TG mice. **F** HPLC analysis of DA content in 7 days postnatal (P7) whole brain and 12 mth midbrain of mice. *P < 0.05, compared with DA content in non-TG mice brains. **G**–**I** Mutant R1441G LRRK2 induces age-relevant alterations of TH and DA levels in TG mice brains. **G** Representative Western blot TH bands in mice cortex. **H** Densitometric analysis of TH bands in Western blot gels in H. *, at least *P* < 0.05, compared with arbitrary value of TH bands in non-TG mice brains. **I** HPLC analysis of DA content in the striatum of 3 month- and 14 month-old mice. *, at least *P* < 0.05, compared with DA content in striatum of non-TG mice. **J**–**L** Age-dependent accumulation of DA-conjugated proteins in non-TG and TG mice midbrains. **J** 12 months, **K** 18 months, **L**, 21 months

We next validated our findings using TG mice models (Fig. 4). We determined age-dependent TH protein levels in G2019S LRRK2 TG mice brains from postnatal 7 days (P7) to 20 months old (Fig. 4A–F, Supplementary Fig. 5). In P7 whole brains and 3 month-old midbrain of G2019S mutant mice, TH protein levels were higher than that of non-transgenic (NTG) control mice (Fig. 4A and B; Supplementary Fig. 5). However, there was a time-dependent decrease in TH protein levels in the midbrain of mutant G2019S mice (Fig. 4C–F). HPLC analysis demonstrated a similar trend of time-dependent changes of DA content (Fig. 4G). These observations were similarly seen in R1441G mutant LRRK2 TG mice (Fig. 4H–I). Increased TH and DA levels were found in younger mice, while a decrease was seen in older ones (Fig. 4H–I). Previous studies have also shown similar trends in G2019S KI mice. Changes in striatal DA levels have not been found in 6-month-old G2019S LRRK2 mice [55, 56], while decreased DA level and DA neuron loss were observed in older 12-, 15- or 18 month- old mutant G2019S mice [55–57]. Interestingly, age-dependent accumulation of DA-conjugated proteins was found in our G2019S mutant mice brains (Fig. 4J–L).

### LRRK2 mutations deregulate TH–DA pathway and DA neuron loss in patient iPSc derived DA neuron and hMLOs models

**Fig. 5:**
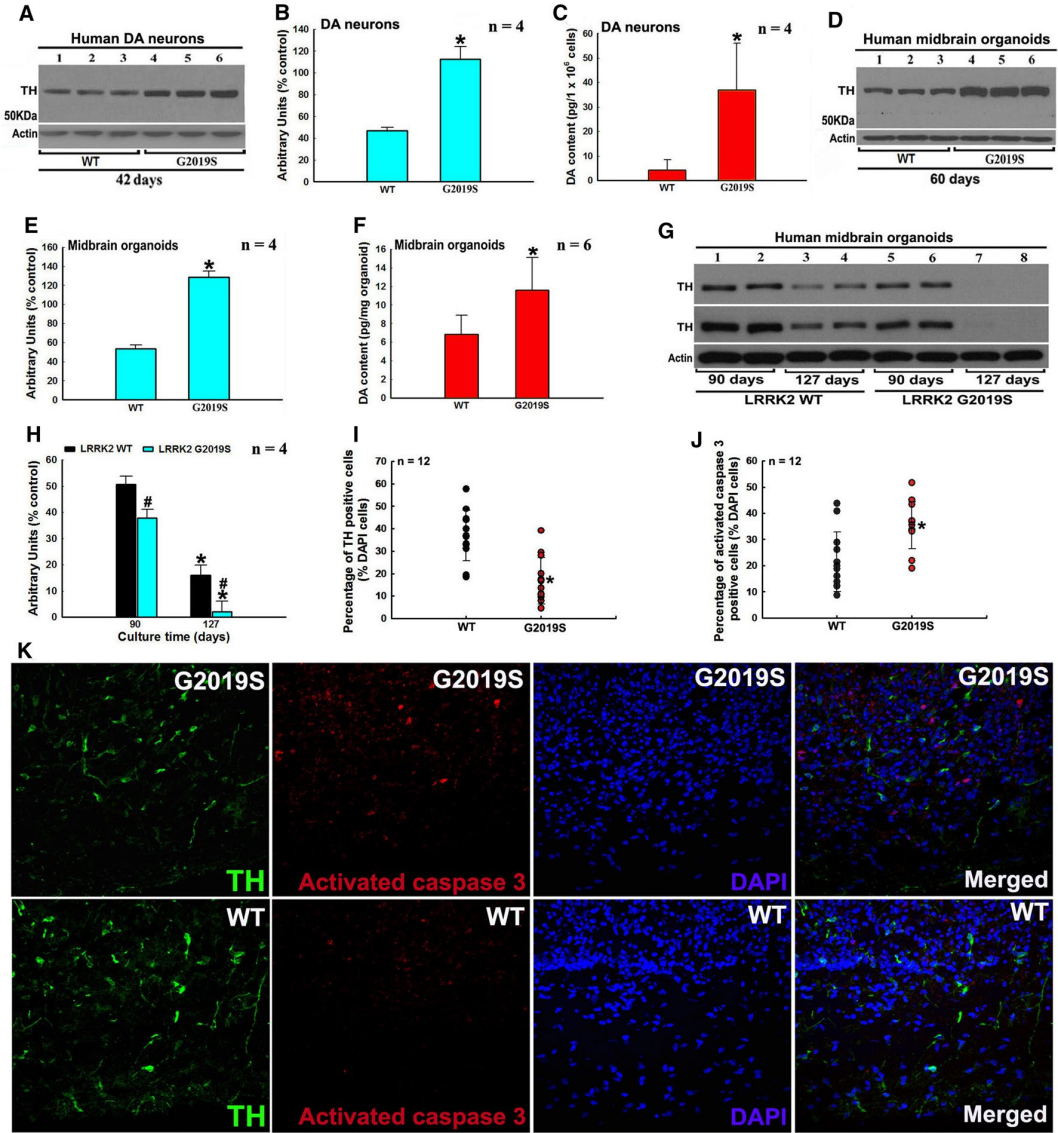
LRRK2 G2019S mutation-induced deregulation of the TH–DA pathway and DA neuron demise in human patient iPSc-derived DA neuron and hMLOs models. **A**–**C** G2019S LRRK2 increases TH and DA levels in iPSc-derived DA neurons after 42 days induction and culture. **A** Representative Western blot data of TH levels in DA neurons. **B** Densitometric analysis of TH protein bands in Western blot gels in A. *P < 0.001, compared with arbitrary value of TH protein band of WT LRRK2 DA neurons, **C** HPLC analysis of DA content in DA neurons. *P < 0.05, compared with DA content in WT LRRK2 DA neurons. **D**–**F** Mutant G2019S LRRK2 increases TH and DA levels in iPSc-derived hMLOs after 60 days induction and culture. **D** Representative Western blot data of TH levels in hMLOs. **E** Densitometric analysis of TH protein bands in Western blot gels in **D**. *P < 0.001, compared with arbitrary value of TH protein bands of WT LRRK2 hMLOs. **F** HPLC analysis of DA content in hMLOs. *P < 0.05, compared with DA content of WT LRRK2 hMLOs. **G** and **H** Mutant G2019S LRRK2 leads to decreased TH level in longer time cultured hMLOs. **G** Western blot TH protein bands in hMLOs after induction and culture for 90 and 127 days. **H** Densitometric analysis of TH bands in Western blot gels in **G**. *P < 0.001, compared with arbitrary value of TH bands of the respective WT or mutant G2019S LRRK2 hMLOs cultured for 90 days. #, at least *P* < 0.05, compared with arbitrary value of TH bands of WT LRRK2 hMLOs cultured for 90 or 127 days, respectively. **I**–**K** Mutant G2019S LRRK2 induces DA neurodegeneration in hMLOs. **I** and **J** Ratio of TH **I** or activated caspase 3- (**J**) positive cells against DAPI-stained cells. *, at least *P *< 0.005, compared with ratio of TH or activated caspase 3-positive cells against DAPI-stained cells of WT LRRK2 hMLOs. **K** Representative confocal image of TH, activated caspase 3 and DAPI-stained hMLOs (magnification: 400×)

We generated pluripotent iPScs using a standard protocol (Supplementary Fig. 6A and B). Subsequently, iPScs were differentiated to generate DA neurons and hMLOs (Supplementary Fig. 6C and D). We found increased TH and DA levels in human G2019S LRRK2 DA neurons (Fig. 5A–C). In hMLOs G2019S model, increased TH and DA levels were found at 60 days, whereas TH and DA levels dropped rapidly with time (90 and 127 days) (Fig. 5D–H). At culture time of 127 days, TH protein was almost undetectable in G2019S LRRK2 hMLOs (Fig. 5G and H). We investigated the ratios of TH and activated caspase 3 positively stained cells among total DAPI-stained cells in G2019S hMLOs and found a decreased ratio of TH-positive cells with increased ratio of activated caspase 3-stained cells at 90 days (Fig. 5I–K). Furthermore, tetracycline-inducible (Tet-on) overexpression of TH in our hMLOs induced DA neurodegeneration (Supplementary Fig. 7). In a recent TH-overexpressing TG mice model, increased TH levels were correlated with increased DA synthesis, elevated ROS generation and reduced glutathione levels [58]. In addition, the TH–HI mice had increased levels of 5-S-cysteinyl-dopamine, a DAQs-cysteine adduction product, in the striatum and the TH–HI mice were more sensitive to stress challenges than control mice [58]. These results support our observations that mutant LRRK2 can deregulate TH–DA pathway, facilitating neuronal death.

### LRRK2 and PINK1 act differently on the TH–DA pathway in *Drosophila*

**Fig. 6:**
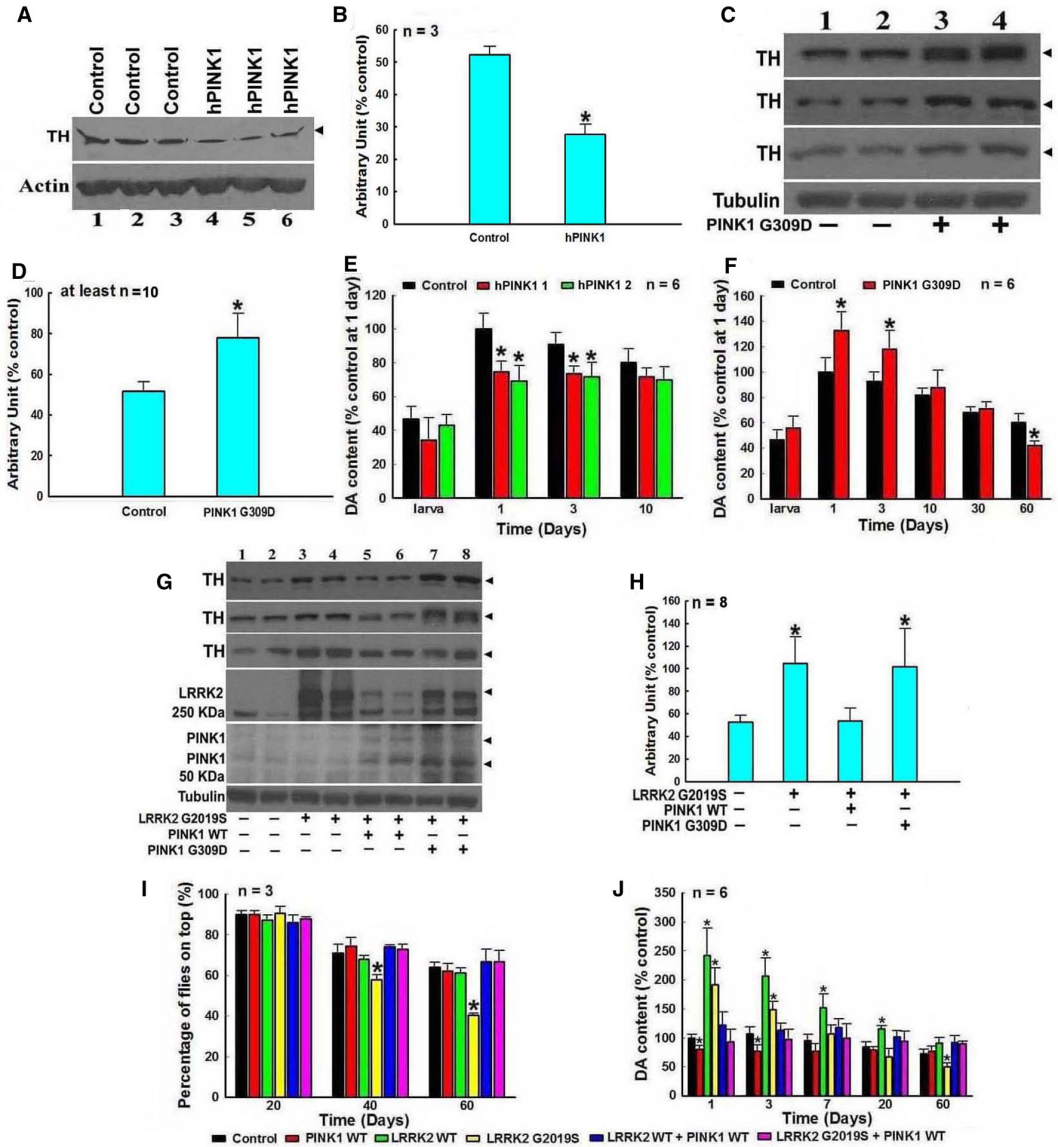
LRRK2 and PINK1 reversely modulate the TH–DA pathway and PD-like symptoms in *Drosophila* PD models. Control yellow white or TG *Drosophila* are crossed with ddc-GAL4 to induce expressions of human PINK1 and/or LRRK2 in DA neurons in *Drosophila* heads. *Drosophila* heads are harvested after 3 days culture, homogenized and subjected to Western blot analysis of TH protein and HPLC analysis of DA content. **A** and **B** WT PINK1 suppresses TH expression in *Drosophila* heads. **A** Western blot data of TH bands in *Drosophila* heads of control and TG WT PINK1 *Drosophila*. **B** Densitometric analysis of TH bands in Western blot gels in A. *, at least *P* < 0.05, compared with the arbitrary value of TH bands in control *Drosophila* heads. **C** and **D** Mutant G309D PINK1 up-regulates TH expression in *Drosophila* heads. **C** Western blot data of TH levels in *Drosophila* heads of control and TG mutant G309D PINK1 *Drosophila*. **D** Densitometric analysis of TH bands in Western blot gels in **C**. *P < 0.05, compared with the arbitrary value of TH bands in control *Drosophila* heads. **E** and **F** HPLC analysis of DA content in *Drosophila* heads of control and TG WT PINK1 (**E**) and mutant G309D (**F**) *Drosophila*. *, at least *P* < 0.05, compared with DA content in *Drosophila* heads of control *Drosophila*. **G**–**J** Regulations of TH expression and DA content in *Drosophila* heads by WT or mutant PINK1 and/or LRRK2. **G** and **H** WT, but not G309D PINK1, can alleviate mutant G2019S LRRK2-induced up-regulation of TH expression level in *Drosophila* heads. **G** Western blot data of TH levels in *Drosophila* heads of control, mutant G2019S LRRK2, mutant G2019S LRRK2 plus WT or mutant G309D PINK1 double TG *Drosophila*. **B** Densitometric analysis of TH bands in Western blot gels in A. *, at least *P* < 0.05, compared with the arbitrary value of TH protein bands in control *Drosophila* heads. **I** WT PINK1 can alleviate mutant G2019S LRRK2-induced PD-like symptoms. *, at least *P* < 0.05, compared with the percentage of *Drosophila* on top of control *Drosophila* cultured for 40 or 60 days, respectively. **J** HPLC analysis of DA content in *Drosophila* heads of control, and transgenic WT and mutant PINK1 and/or LRRK2 *Drosophila* lines. *, at least *P* < 0.05, compared with DA content in *Drosophila* heads of control *Drosophila* cultured for the respective days

We posit that PINK1 and LRRK2 may modulate the TH–DA pathway in DA neurons differentially. Double TG *Drosophila* lines with WT or mutant LRRK2 and PINK1 were generated and we found that WT PINK1 down-regulated, whereas mutant G309D PINK1 up-regulated TH and DA levels in TG *Drosophila*. (Fig. 6A–F). Furthermore, WT PINK1, but not G309D PINK1, suppressed G2019S-induced TH increase at 3 days of culture. Co-expression of WT PINK1 and G2019S mutant LRRK2 inhibited the early increase of TH and DA levels and improved PD-like phenotype and subsequent DA level (Fig. 6G–J). Separately, we also found that DJ-1 can protect DA neurons, but the pattern of changes found in LRRK2 and PINK1 *Drosophila* models was not observed (Supplementary Fig. 8).

### LRRK2 and PINK1 suppress expression reciprocally in different PD models

**Fig. 7:**
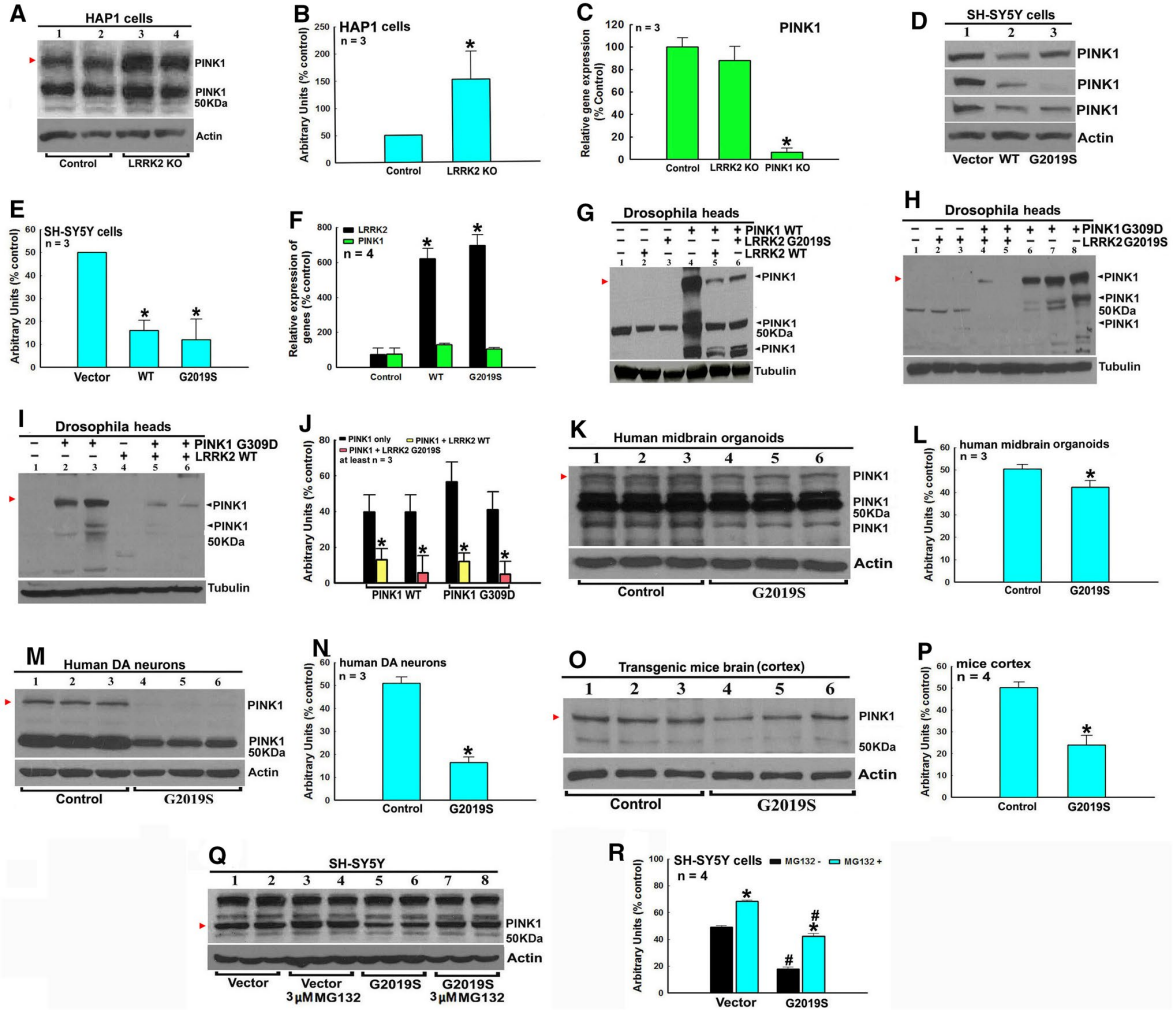
LRRK2 down-regulates PINK1 protein level via facilitation of PINK1 protein degradation in an LRRK2 kinase activity-dependent manner. **A**–**C** LRRK2 KO up-regulates PINK1 protein level in human HAP1 cells. **A** Representative Western blot data of PINK1 levels in LRRK2 KO HAP1 cells. **B** Densitometric analysis of red arrowhead-pointed PINK1 bands in Western blot gels in **A**. *, *P* < 0.01, compared with the arbitrary value of PINK1 bands in control HAP1 cells. **C** LRRK2 KO has no influence on PINK1 transcription level in HAP1 cells, demonstrated by quantitative real-time RT-PCR analysis. *P < 0.001, compared with the expression level of PINK1 in control HAP1 cells. **D**–**F** Stable transfection of WT and mutant G2019S LRRK2 down-regulates PINK1 protein level in SH-SY5Y cells. **D** Western blot data of PINK1 bands in empty vector, WT or mutant G2019S LRRK2 stably transfected cells. **E** Densitometric analysis of red arrowhead-pointed PINK1 bands in Western blot gels in **D**. *, *P* < 0.001, compared with the arbitrary value of PINK1 bands in empty vector transfected cells. **F** Quantitative real-time RT-PCR analysis showed that stable transfection of WT and mutant G2019S LRRK2 had no impact on PINK1 transcription level in cells. *P < 0.001, compared with LRRK2 transcription level of empty vector transfected cells. **G**–**J** Human WT and mutant G2019S LRRK2 down-regulate PINK1 protein level in LRRK2/PINK1 double TG *Drosophila* heads. Single or double TG LRRK2 and/or PINK1 lines are crossed with ddc-GAL4 line to induce expressions of PINK1 and or LRRK2 in DA neurons in *Drosophila* heads. *Drosophila* heads are harvested after 3 days culture, homogenized and subjected to Western blot analysis of PINK1 protein level. **G**–**I** Representative Western blot data of PINK1 level in *Drosophila* heads of control, WT or G309D PINK1, WT or mutant G2019S LRRK2 single or double TG *Drosophila*. **G** WT PINK1 and/or WT or mutant G2019S LRRK2; **H** mutant G309D PINK1 and/or mutant G2019S LRRK2 *Drosophila*. **I** Mutant G309D PINK1 and or WT LRRK2 *Drosophila*. **J** Densitometric analysis of red arrowhead-pointed PINK1 protein bands in Western blot gels in **G**, **H** and **I**. *, at least *P* < 0.05, compared with the arbitrary value of PINK1 bands in single WT or mutant G309D PINK1 TG *Drosophila*. **K** and **L** Mutant G2019S LRRK2 down-regulates PINK1 protein level in iPSc derived hMLOs. **K** Western blot data of PINK1 levels in hMLOs with mutant G2019S LRRK2 after 60 days induction and culture. **L** Densitometric analysis of red arrowhead-pointed PINK1 bands in Western blot gels in J. *, *P* < 0.05, compared with the arbitrary value of PINK1 bands of hMLOs with WT LRRK2. **M** and **N**, mutant G2019S LRRK2 down-regulates PINK1 protein level in iPSc derived human DA neurons. **M** Western blot data of modulated PINK1 levels in human DA neurons with mutant G2019S LRRK2 after 42 days induction and culture. **N** Densitometric analysis of red arrow head pointed PINK1 bands in Western blot gels in L. *P < 0.0001, compared with the arbitrary value of PINK1 protein bands of human DA neurons with WT LRRK2. **O** and **P** Mutant G2019S LRRK2 down-regulates PINK1 protein level in mutant G2019S LRRK2 TG mice cortex. **O** Representative Western blot data of modulated PINK1 protein levels in 6 month aged TG mice cortex by mutant G2019S LRRK2. **P** Densitometric analysis of red arrow head pointed PINK1 bands in Western blot gels in O. *P < 0.001, compared with the arbitrary value of PINK1 protein bands of control non-transgenic mice. **Q**–**R** Mutant G2019S LRRK2 promotes proteasome degradation of PINK1 protein in SH-SY5Y cells. **Q** Representative Western blot data of PINK1 levels in empty vector or mutant G2019S LRRK stably transfected cells in the presence or absence of 3 µM proteasome inhibitor MG132 for 6 h. **R** Densitometric analysis of red arrowhead-pointed PINK1 bands in Western blot gels in **Q**. *, at least *P* < 0.001, compared with the arbitrary value of PINK1 bands of respective cells without MG132 treatment. #, at least *P* < 0.001, compared with the arbitrary value of PINK1 bands of empty vector transfected cells with or without MG132 treatment, respectively

PINK1 can be proteolytically cleaved into multiple fragments (Fig. 7). We observed different expression and proteolytical cleavage patterns of PINK1 proteins in *Drosophila*, mice and human cell and tissue models (Fig. 7). We found that LRRK2 KO in human HAP1 cells increased PINK1 protein level with no influence on PINK1 transcription level (Fig. 7A–C). PINK1 protein level, but not PINK1 transcription, was decreased in WT and mutant G2019S LRRK2 SH-SY5Y stable cells (Fig. 7D–F). In HEK cells, overexpression of G2019S, but not WT or 3KD, RCK domains of LRRK2 decreased PINK1 protein level (Supplementary Fig. 9). Furthermore, overexpression of WT or G2019S LRRK2 down-regulated WT or G309D PINK1 levels in double transgenic *Drosophila* models (Fig. 7G–J). PINK1 protein levels were decreased in iPSc-derived LRRK2 G2019S mutant human DA neurons and hMLOs (Fig. 7K–N). PINK1 protein levels were decreased in TG G2019S mice (Fig. 7O–P). However, inhibition of proteasome function by MG132 alleviated LRRK2-induced PINK1 protein level decrease in SH-SY5Y cells, suggesting that LRRK2 promotes proteasome degradation of PINK1 protein (Fig. 7Q and R).

**Fig. 8:**
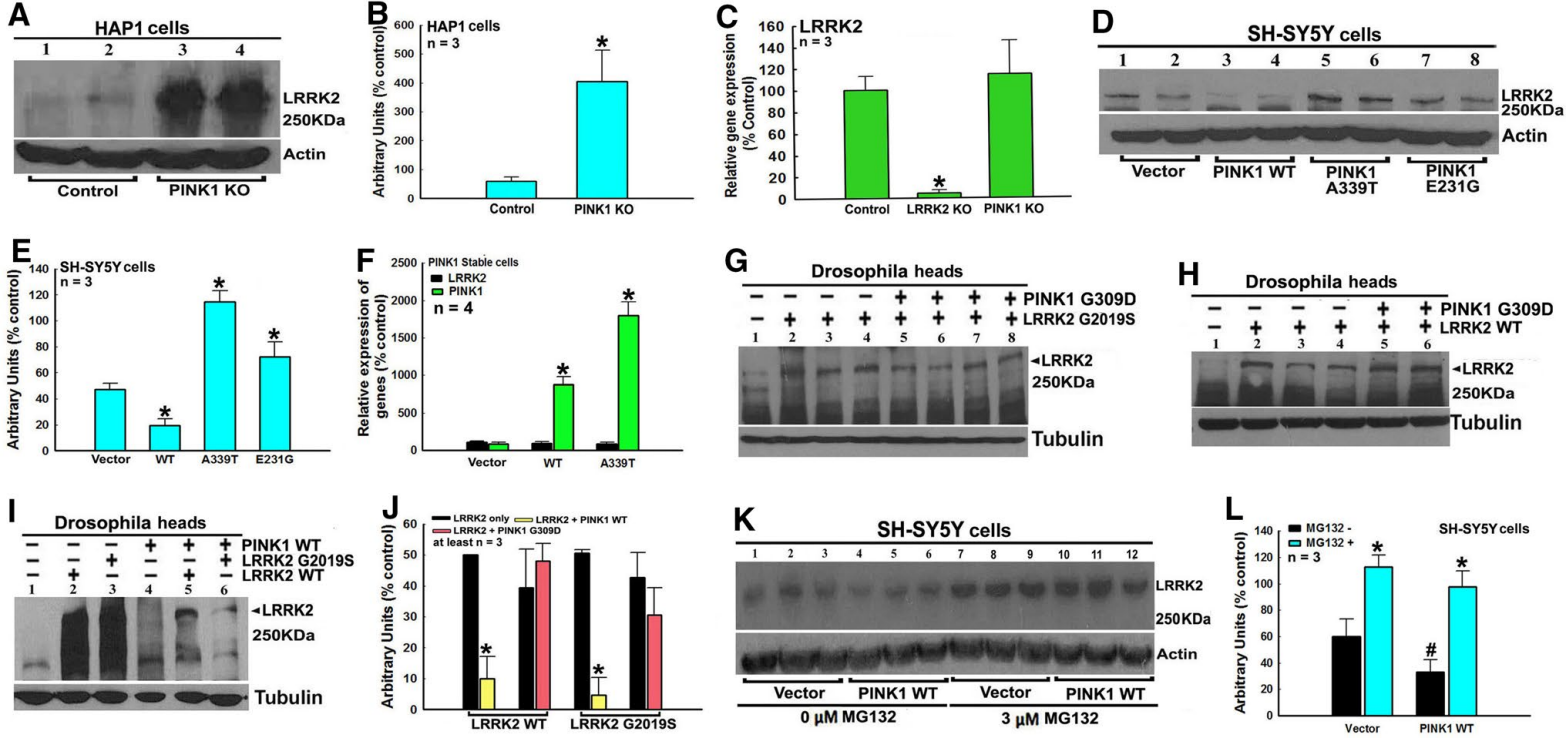
PINK1 down-regulates LRRK2 protein level via facilitation of LRRK2 protein degradation in a PINK 1 kinase activity-dependent manner. **A**–**C** PINK1 KO up-regulate LRRK2 protein level in human HAP1 cells. **A** Representative Western blot data of LRRK2 levels in PINK1 KO HAP1 cells. **B** Densitometric analysis of LRRK2 bands in Western blot gels in **A**. *P < 0.01, compared with the arbitrary value of LRRK2 bands in control HAP1 cells. **C** PINK1 KO has no influence on LRRK2 transcript level in HAP1 cells. *P < 0.001, compared with the expression level of PINK1 in control HAP1 cells. **D**–**F** Stable transfection of WT PINK1 down-regulate LRRK2 protein level, whereas stable transfection of mutant A339T or E231G PINK1 up-regulate LRRK2 protein level in SH-SY5Y cells. **D** Representative Western blot data of LRRK2 level in empty vector, WT or mutant A339T or E231G PINK1 stably transfected cells. **E** Densitometric analysis of LRRK2 bands in Western blot gels in D. *, at least *P* < 0.05, compared with the arbitrary value of LRRK2 protein bands in empty vector transfected cells. **F** Quantitative real-time RT-PCR analysis of PINK1 and LRRK2 expression in stably transfected cells. *, at least *P* < 0.001, compared with PINK1 transcript level of empty vector transfected cells. **G**–**J** WT, but not mutant G309D PINK1 down-regulate LRRK2 protein level in double TG *Drosophila* heads. Single or double TG WT or mutant LRRK2 and/or PINK1 lines are crossed with ddc-GAL4 line to induce expressions of PINK1 and/or LRRK2 in DA neurons in *Drosophila* heads. *Drosophila* heads are harvested after 3 days culture, homogenized and subjected to Western blot analysis of PINK1 protein level. **G**–**I** Representative Western blot data of LRRK2 bands in *Drosophila* heads of control, single or double TG *Drosophila*. **G** Mutant G309D PINK1 and/or mutant G2019S LRRK2 *Drosophila*; **H** mutant G309D PINK1 and/or WT LRRK2 *Drosophila*; **I** WT PINK1 and/or WT or mutant G2019S LRRK2 *Drosophila*. **J** Densitometric analysis of LRRK2 bands in Western blot gels in **G**, **H** and **I**. *, at least *P* < 0.01, compared with the arbitrary value of LRRK2 bands in *Drosophila* heads of WT or mutant G2019S LRRK2 single TG *Drosophila*. (**K** and **L**) WT PINK1 promotes proteasome degradation of LRRK2 protein in SH-SY5Y cells. **K** Representative Western blot data of LRRK2 levels in empty vector or WT PINK1 stably transfected cells treated with or without 3 µM MG132 for 6 h. **L** Densitometric analysis of LRRK2 bands in Western blot gels in **K**. *, at least *P* < 0.05, compared with the arbitrary value of LRRK2 bands of respective cells without MG132 treatment. ^#^*P* < 0.05, compared with the arbitrary value of LRRK2 bands of empty vector transfected cells without MG132 treatment

In PINK1 KO HAP1 cells, LRRK2 protein level was up-regulated with no effect on LRRK2 transcription (Fig. 8A–C). WT PINK1, but not mutant A339T and E231G PINK1, down-regulated LRRK2 protein level in PINK1 stable SH-SY5Y cells with no effects on LRRK2 transcription (Fig. 8D–F). WT PINK1, but not G309D PINK1, down-regulated WT or G2019S LRRK2 protein levels in *Drosophila* (Fig. 8G–J). However, MG132 treatment reversed WT PINK1-induced down-regulation of LRRK2 protein level in SH-SY5Y cells (Fig. 8K and L).

**Scheme 1:**
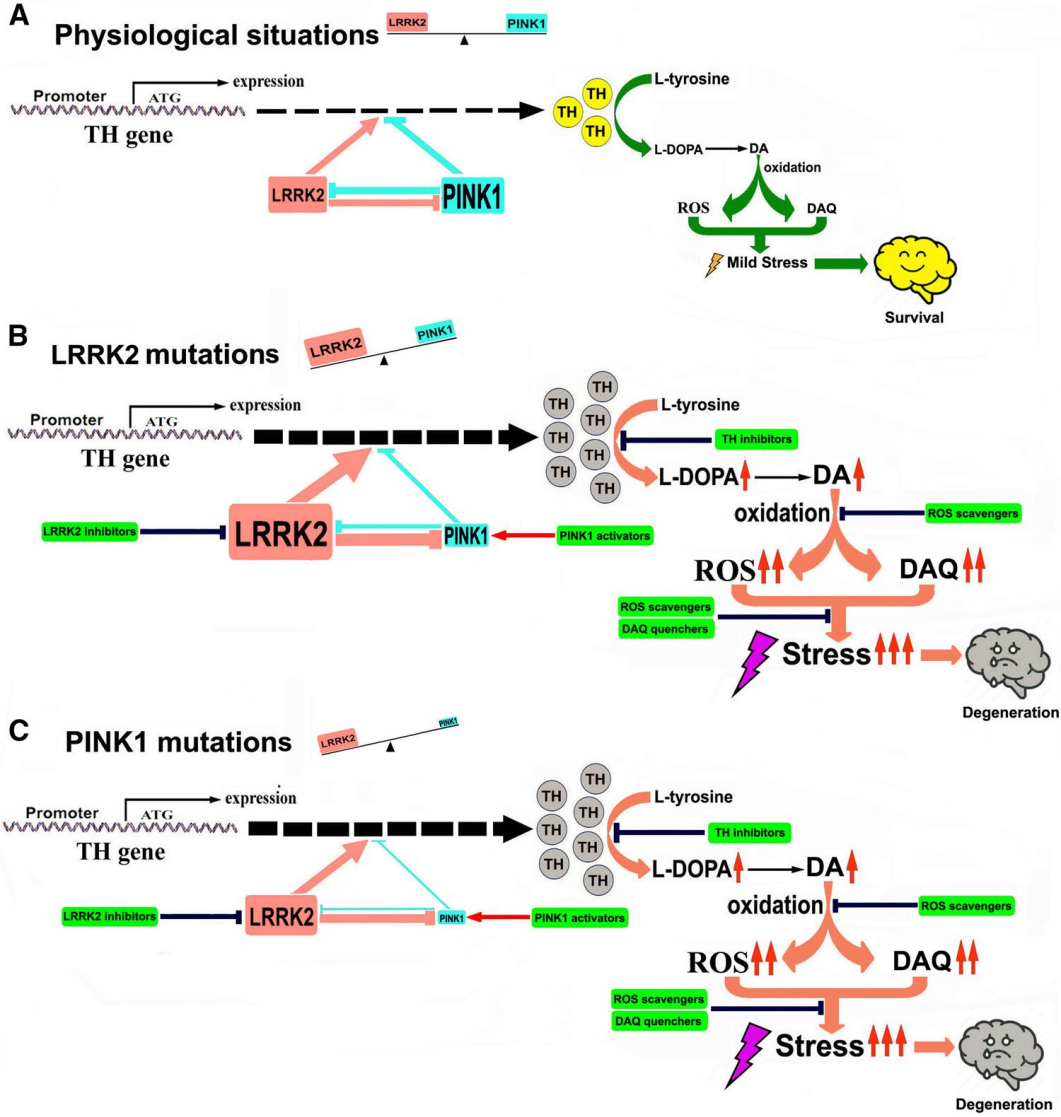
Illustration of the role of LRRK2–PINK1 on TH expression and DA synthesis in DA neurons. Under physiological conditions, LRRK2 and PINK1 form a functional balance to maintain normal TH expression and DA synthesis in DA neurons. LRRK2 promotes TH expression and DA generation, while PINK1 suppresses TH expression and DA generation. LRRK2 and PINK1 can regulate degradation of each other, and thus a balance can be reached. When LRRK2 is mutated, its kinase activity is increased, leading to up-regulated TH expression and increased DA generation. Increased LRRK2 kinase activity can facilitate PINK1 degradation, down-regulate PINK1 level and suppress PINK1 function. This will lead to imbalance between LRRK2 and PINK1, contributing to increased TH expression, enhanced DA generation, aggravated DA oxidation and elevated DA-relevant stress in DA neurons, promoting neurodegeneration. When PINK1 is mutated, kinase activity will be impaired causing LRRK2–PINK1 imbalance and disrupting the TH–DA pathway, promoting DA neuron vulnerability and neurodegeneration

Taken together, PINK1 and LRRK2 have opposing effects on TH expression and the TH–DA pathway, thereby influencing DA neuron survival. LRRK2 or PINK1 mutations disrupt the physiologic balance, facilitating DA toxicity and neurodegeneration (Scheme 1).

## Discussion

Using PD *Drosophila*, mice and human neuron and hMLOs models, we found that LRRK2 mutations cause an up-regulation of TH expression and DA levels at the early stage of disease and this promotes DA neuron degeneration. Enhanced TH expression has been found in stress-induced DA neuron loss in vitro [26], and TG overexpression of TH in mice brains can lead to increased DA synthesis, elevated DA toxicity and neurodegeneration [58]. Tet-on induced TH overexpression in hMLOs-induced DA neurodegeneration (Supplementary Fig. 7). However, RNAi knockdown of TH expression significantly protected against rotenone and mutant α-syn-induced DA neurodegeneration in *Drosophila* [30]. Furthermore, endogenous DA can be a predisposing factor, as deleterious DA oxidative metabolites promote selective DA neurodegeneration in PD [27–35, 37–40, 42, 44, 45, 59–64]. Injection of DA into rat striatum induces gliosis and DA neurodegeneration in rat brain [65]. In our in vitro and in vivo models, we also found that inhibition of TH by α-MT and anti-oxidative GSH treatment reversed DA toxicity and mutant LRRK2-induced DA neurodegeneration. These highlight the importance of DA homeostasis in maintaining the integrity of DA neurons and their responses to stress challenges. Therapeutic strategies focusing on TH–DA pathway to modulate TH activity and therapeutic approaches to counteract DA toxicity will be neuroprotective.

It will be interesting to investigate whether up-regulated TH–DA pathway functions can be identified in early stage of high-risk populations, such as LRRK2 or PINK1 mutation healthy carriers. The DA system in human brains can be visualized and quantitatively analyzed by advanced molecular functional magnetic resonance imaging (MRI) techniques. Positron emission tomography (PET) and single photon emission computed tomography (SPECT) techniques can quantitatively monitor and visualize different aspects of the DA system to improve the accuracy of the diagnosis, including DA receptors, DA transporters, and DA release in the brain [66, 67], whereas the novel neuromelanin (NM)-sensitive MRI (NM-MRI) can visualize and semi-quantify NM in SN of human brains [66]. The NM is the end product of DA oxidation, which is formed via polymerization of DA-derived DAQ [45]. Increased NM accumulation in brains indicates enhanced DA generation and DA turnover in DA neurons, suggesting aggravated DA toxicity in SN. Therefore PET, SPECT and NM-MRI are potentially useful to screen and monitor the DA system and NM level in brains of human subjects, especially high-risk cohorts. Healthy subjects identified with increased DA functions and NM signals can be selected for neuroprotective drug trials.

Although significant advancements have been made to alleviate PD symptoms over the past few years, including DA-based medications, deep brain stimulation and rehabilitative therapies, the progressive DA neuron loss in PD patient brains cannot be reversed [68]. In the current study, we showed that inhibition of TH by continuous low-dose α-MT administration initiated at the early stage was able to prevent LRRK2 G2019S mutation-induced DA neurodegeneration in our PD models. α-MT is an orally competitive TH inhibitor, which has been used clinically to treat hypertension-linked pheochromocytoma and dystonia, dyskinesia and Huntington's disease [53, 54, 69]. Low-dose α-MT has been shown to be safe with no significant side effects even after prolonged use (3 years) [54]. Considering the excellent neurological pharmacology features with low toxicity and high human subject tolerance, low-dosage TH inhibitor therapy with α-MT seems to be a promising approach to protect DA neurons and prevent PD neurodegeneration. Early clinical trials in LRRK2 asymptomatic carriers can be a consideration.

LRRK2 and PINK1 regulate cellular vesicle trafficking, p53 apoptotic pathway and mitophagy process [24, 70–74]. In the current study, we found an interesting reciprocal function of LRRK2 and PINK1 on TH expression. It is tempting to speculate that LRRK2 and PINK1 may act as a functional protein kinase pair to regulate the TH–DA pathway in an antagonistic manner in DA neurons. LRRK2 facilitates, whereas PINK1 suppresses TH expression and DA generation in an LRRK2 and PINK1 kinase activity-dependent manner (Scheme 1). Furthermore, LRRK2 and PINK1 facilitate proteasome degradation of PINK1 and LRRK2 proteins to reciprocally suppress their functions (Scheme 1A). An LRRK2–PINK1 physiologic balance in the TH–DA pathway may be important for DA neuron viability. Under normal conditions, WT LRRK2 and PINK1 act together to maintain physiological TH and DA levels and promote DA neuron survival. However, when LRRK2 or PINK1 is mutated, the LRRK2–PINK1 balance will be disturbed (Scheme 1B and C). PD-linked LRRK2 mutations up-regulated TH and DA levels. The toxicity of different PD-linked LRRK2 mutants can be dependent on enhanced LRRK2 kinase activity [15, 75]. LRRK2 mutations facilitate UPS degradation of PINK1 protein to inhibit PINK1 functions (Scheme 1B). The enhanced LRRK2 functions and suppressed PINK1 activities under LRRK2 mutations caused LRRK2–PINK1 imbalance, leading to deregulation of TH–DA pathway, DA toxicity and DA neuron vulnerability (Scheme 1B). On the other hand, PD-linked PINK1 mutations have decreased kinase activity and impair PINK1 inhibition of TH expression (Scheme 1C). PINK1 mutations impair UPS degradation of LRRK2 protein (Scheme 1C). The down-regulated PINK1 activity and relatively up-regulated LRRK2 function as a result of PINK1 mutation disrupt LRRK2–PINK1 imbalance, contributing to deregulated TH–DA pathway and DA neuron vulnerability (Scheme 1C).

Multiple factors, including transcription factors such as Pitx3, Nurr1, CREB, ATF2, CREM-1 and NRSF, can regulate TH expression [76, 77]. Other proteins, such as metastasis-associated protein 1 and heterogeneous nuclear ribonucleoprotein K, bind to TH gen promoter to stimulate TH transcription in neuronal cells [78, 79]. TH expression can also be modulated via complex mechanisms involving aryl hydrocarbon receptor, histone H3 acetylation and DA transporter [80–82]. The potential role of LRRK2–PINK1 kinase pair to interact with these TFs, proteins and pathways to modulate TH expression needs to be further investigated. TH can be phosphorylated by protein kinases at Ser31 (Serine31) or Ser40 (Serine40) to enhance TH activity in DA synthesis [83]. Future studies on potential direct interactions of LRRK2 or PINK1 with TH protein to modulate TH functions via phosphorylation regulation will be interesting.

## Conclusions

In summary, using PD *Drosophila*, mice and hMLOs models, we demonstrated that LRRK2 mutations up-regulate TH expression and DA levels at the early stage of disease and this led to DA toxicity and facilitated DA neuron degeneration. Inhibition of TH by a clinical-grade drug, α-MT, at the early stage was able to prevent LRRK2 mutation-induced DA neurodegeneration in our PD models. We also identified a reciprocal function of LRRK2 and PINK1 on TH expression, suggesting that they may potentially act as a functional protein kinase pair to regulate TH–DA pathway. LRRK2 or PINK1 mutations can disrupt this balance, promoting DA neuron demise. Our findings provide support for potential clinical trials using the TH–DA pathway inhibitors in early or prodromic PD.

## Supplementary Information

Below is the link to the electronic supplementary material.

Supplementary file1 (JPG 398 KB)

Supplementary file2 (JPG 144 KB)

Supplementary file3 (JPG 234 KB)

Supplementary file4 (JPG 120 KB)

Supplementary file5 (JPG 180 KB)

Supplementary file6 (JPG 1684 KB)

Supplementary file7 (JPG 717 KB)

Supplementary file8 (JPG 233 KB)

Supplementary file9 (JPG 182 KB)

Supplementary file10 (DOCX 14 KB)

Supplementary file11 (DOCX 13 KB)

Supplementary file12 (DOCX 13 KB)

Supplementary file13 (DOCX 13 KB)

Supplementary file14 (DOCX 12 KB)

Supplementary file14 (DOCX 12 KB)

Supplementary file14 (DOCX 18 KB)

## Data Availability

The datasets generated and/or analyzed in this study are available from the corresponding author Zhou Zhi Dong on request.

## Abbreviations

α-MT: α-Methyl-tyrosine
α-syn: α-Synuclein
BDNF: Brain-derived neurotrophic factor
COR: C-terminal of ROC
DA: Dopamine
DAQs: Dopamine quinones
DMEM: Dulbecco’s modified Eagle medium
DNP: Dinitrophenylhydrazone
DOPAL: 3,4-Dihydroxyphenylacetaldehyde
EBs: Embryoid bodies
ESC: Embryonic stem cell
FGF2: Fibroblast growth factor 2
FGF8: Fibroblast growth factor 8
GDNF: Glial cell-derived neurotrophic factor
hMLOs: Human midbrain-like organoids
HPLC: High-performance liquid chromatography
IACUC: Institutional Animal Care and Use Committee
iPSCs: Induced pluripotent stem cells
LRRK2: Leucine-rich repeat kinase 2
MRI: Magnetic resonance imaging
MTT: 3-(4,5-Dimethylthiazol-2-yl)-2,5-diphenyltetrazolium bromide
NM: Neuromelanin
NM-MRI: Neuromelanin-sensitive MRI
NNI: National Neuroscience Institute
NEAA: Nonessential amino acids
NPC: Neural progenitor cells
PCR: Polymerase chain reaction
PD: Parkinson’s disease
PET: Positron emission tomography
PINK1: PTEN-induced kinase 1
PTEN: Phosphatase and tensin homolog deleted on chromosome 10
ROC: Ras-of-complex proteins
ROS: Reactive oxygen species
RT-PCR: Reverse transcription PCR
SDS: Sodium dodecyl sulfate
SHH: Sonic hedgehog
SN: Substantia nigra pars compacta
SPECT: Single-photon emission computed tomography
Tet-on: Tetracycline-inducible
TG: Transgenic
TH: Tyrosine hydroxylase
TTSH: Tan Tock Seng Hospital
UPS: Ubiquitin–proteasome system
WT: Wild type
