# Supplementary figures and images for: The role of tyrosine hydroxylase–dopamine pathway in Parkinson’s disease pathogenesis
Source: Cell Mol Life Sci. 2022 Nov 21;79(12):599. doi: 10.1007/s00018-022-04574-x (PMC9678997; doi:10.1007/s00018-022-04574-x)

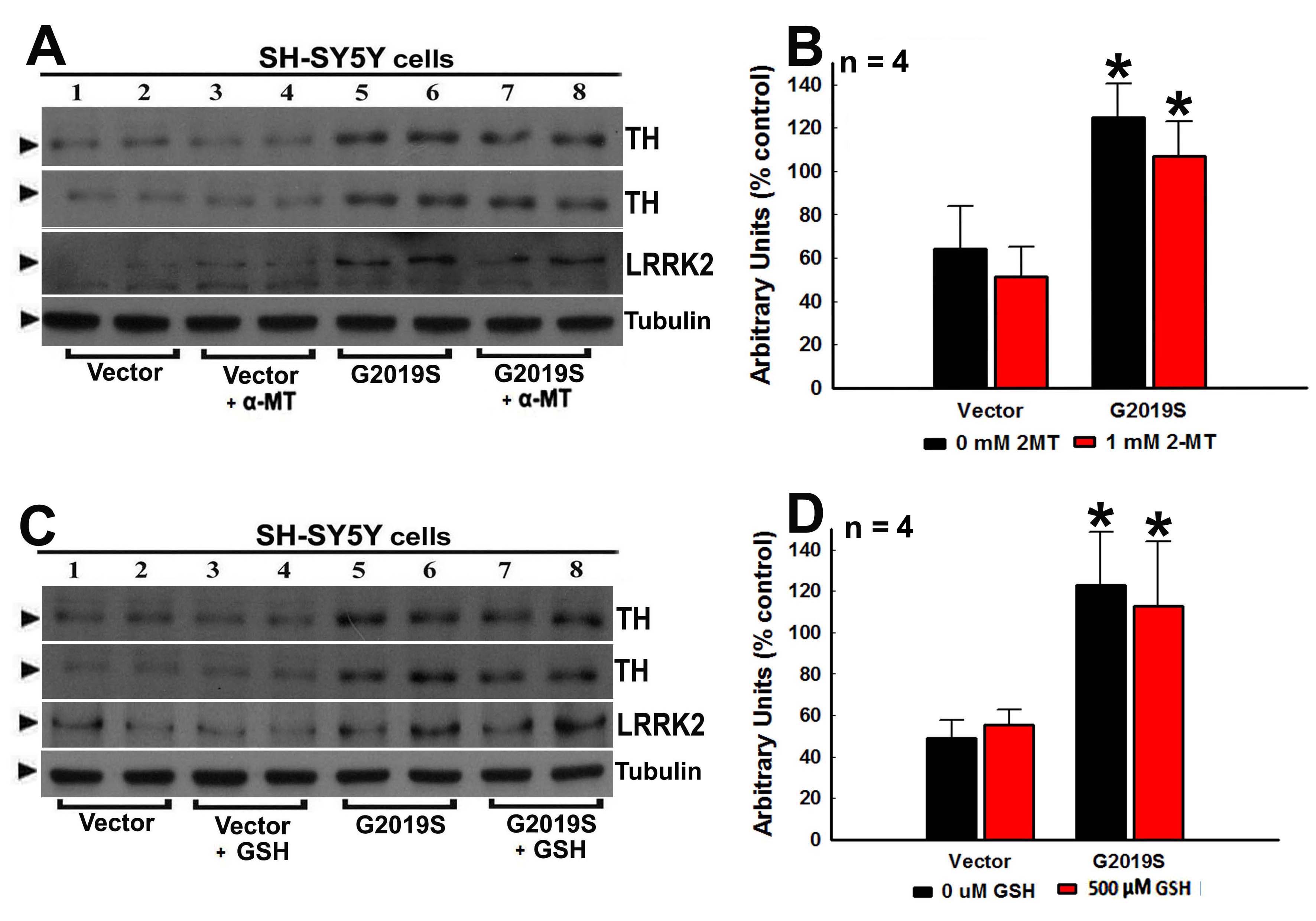

Supplement: Supplementary file 1 — Supplementary file1 (JPG 398 KB) [file 18_2022_4574_MOESM1_ESM.jpg]

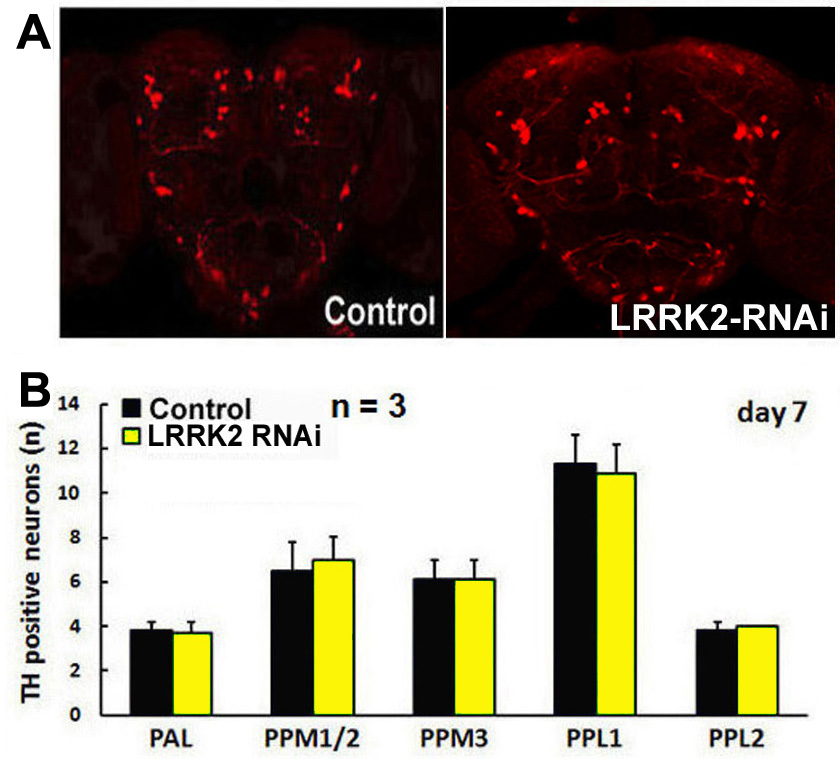

Supplement: Supplementary file 2 — Supplementary file2 (JPG 144 KB) [file 18_2022_4574_MOESM2_ESM.jpg]

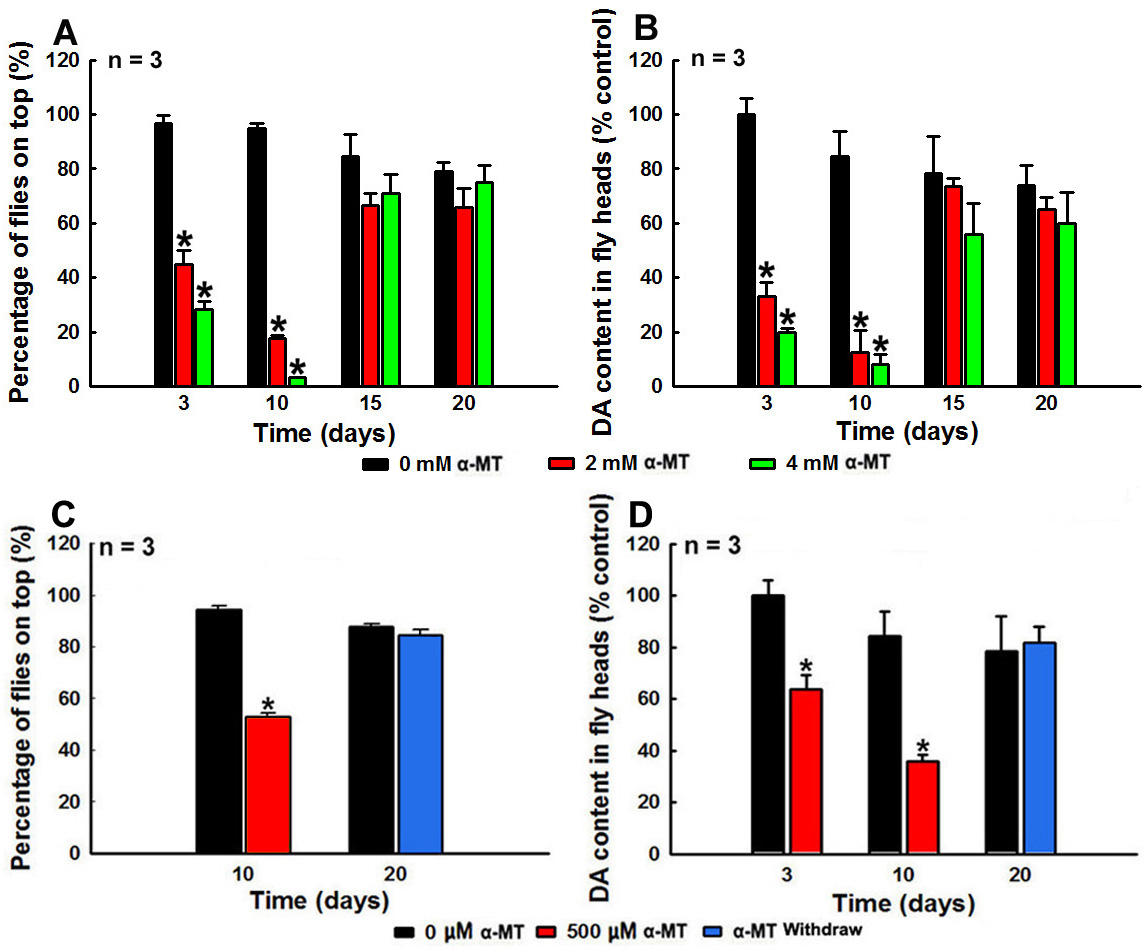

Supplement: Supplementary file 3 — Supplementary file3 (JPG 234 KB) [file 18_2022_4574_MOESM3_ESM.jpg]

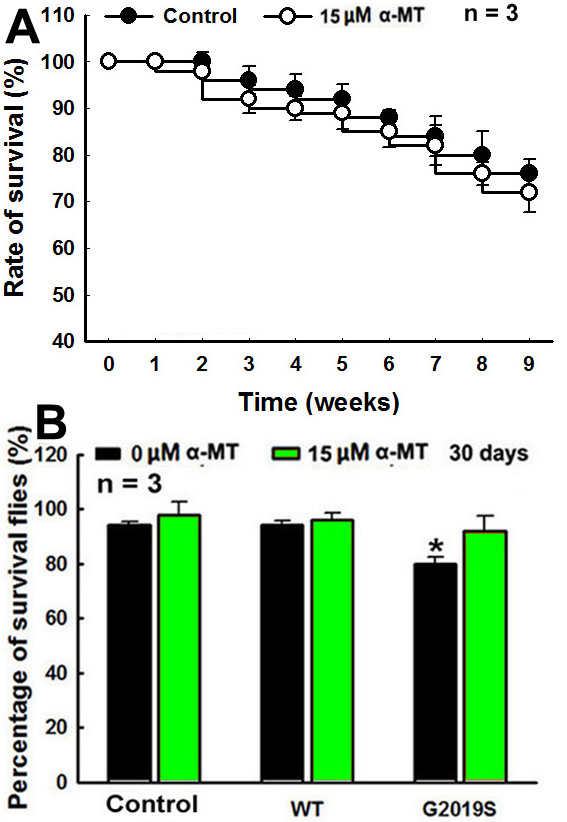

Supplement: Supplementary file 4 — Supplementary file4 (JPG 120 KB) [file 18_2022_4574_MOESM4_ESM.jpg]

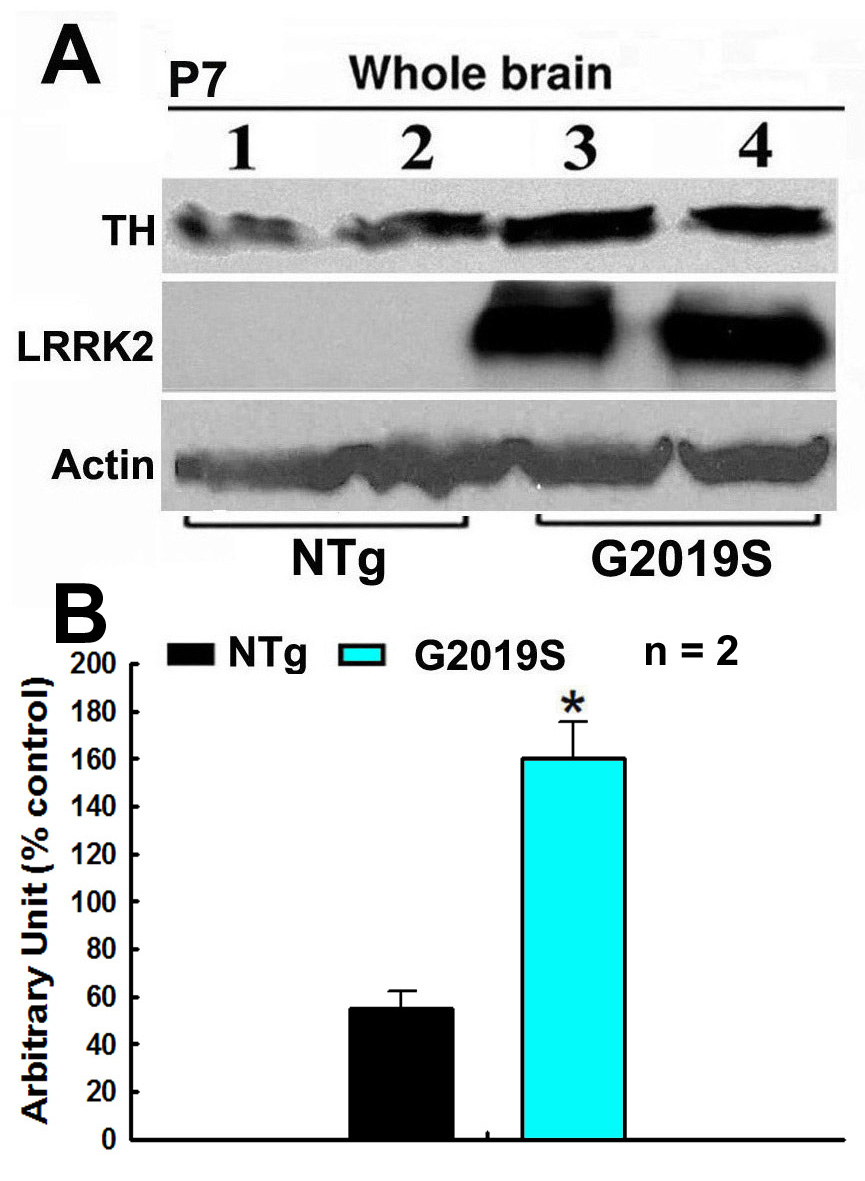

Supplement: Supplementary file 5 — Supplementary file5 (JPG 180 KB) [file 18_2022_4574_MOESM5_ESM.jpg]

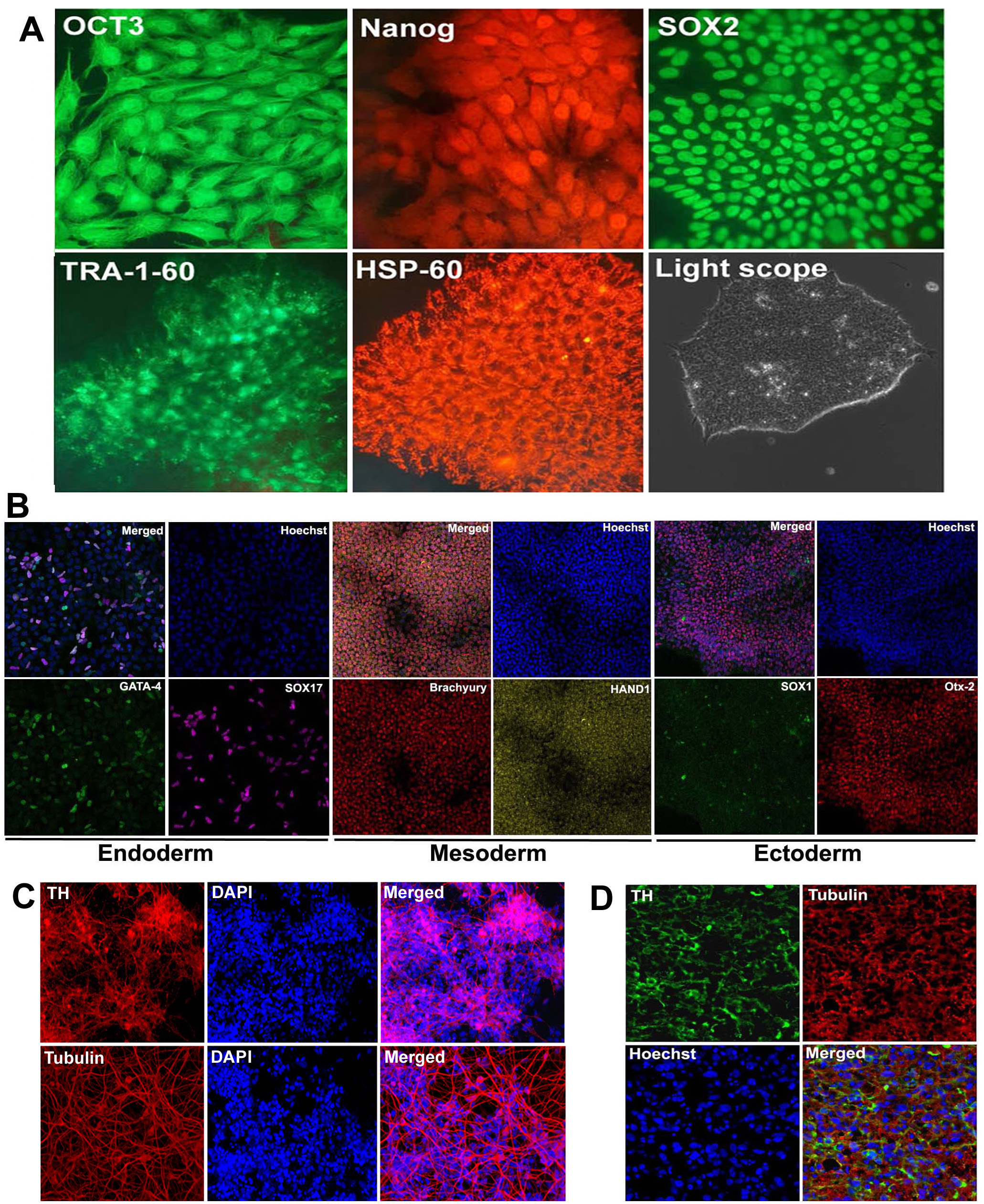

Supplement: Supplementary file 6 — Supplementary file6 (JPG 1684 KB) [file 18_2022_4574_MOESM6_ESM.jpg]

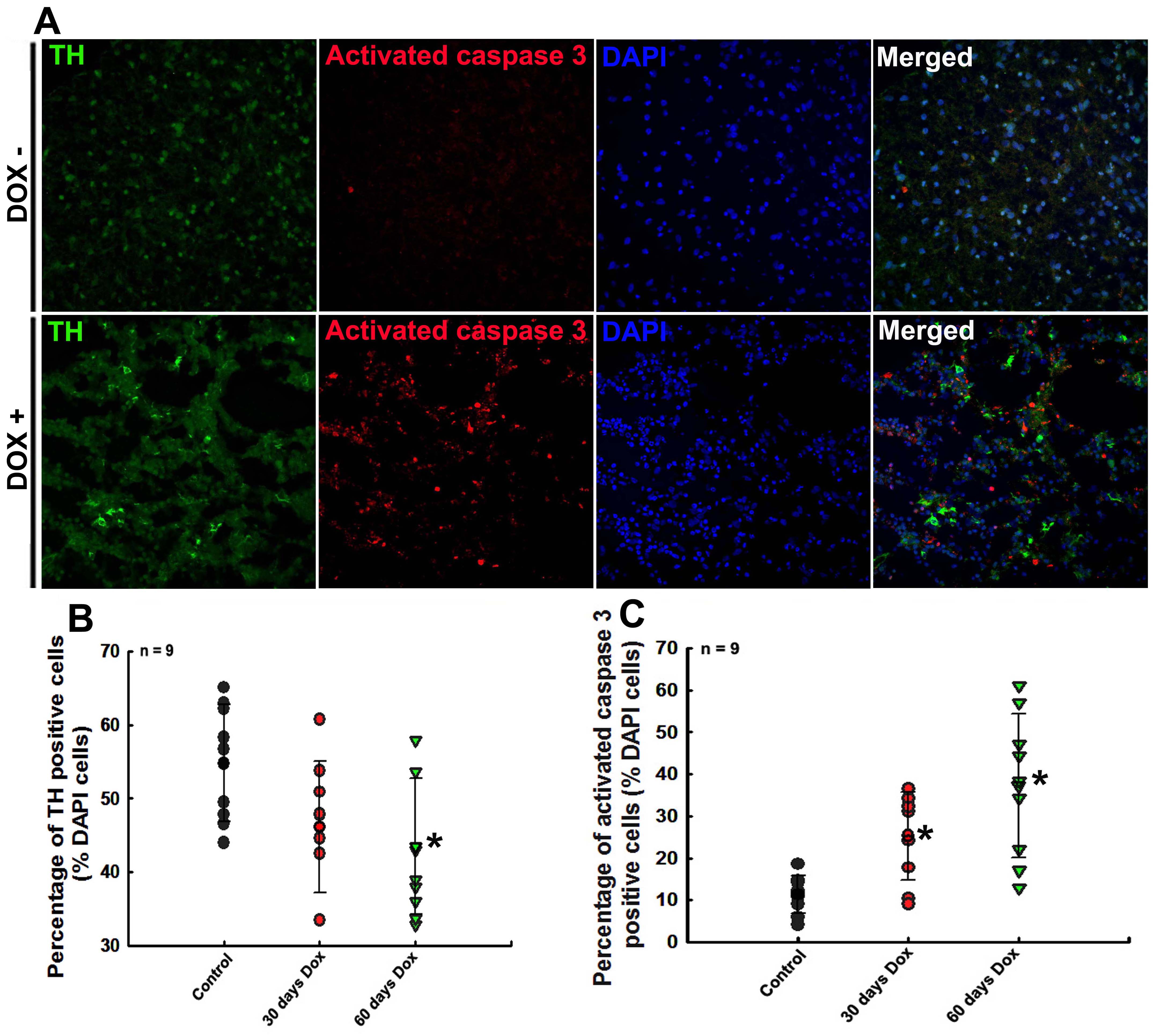

Supplement: Supplementary file 7 — Supplementary file7 (JPG 717 KB) [file 18_2022_4574_MOESM7_ESM.jpg]

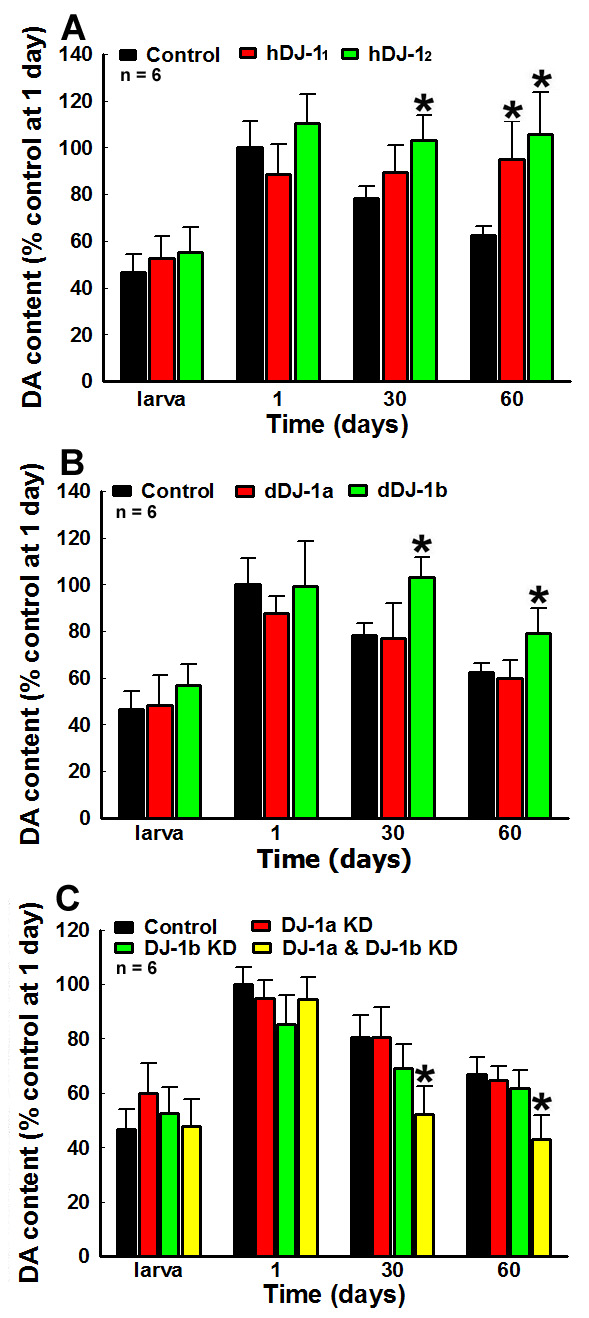

Supplement: Supplementary file 8 — Supplementary file8 (JPG 233 KB) [file 18_2022_4574_MOESM8_ESM.jpg]

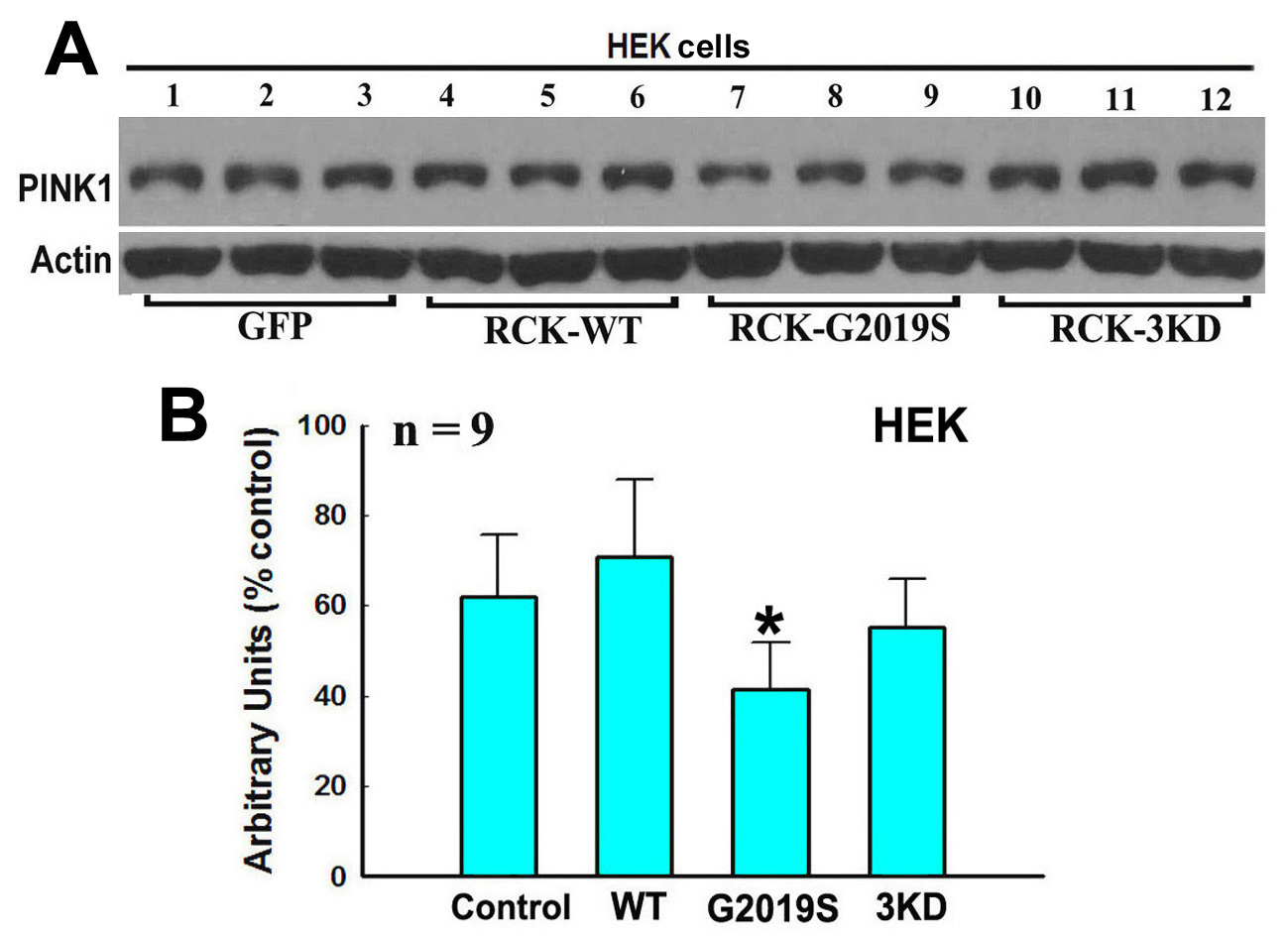

Supplement: Supplementary file 9 — Supplementary file9 (JPG 182 KB) [file 18_2022_4574_MOESM9_ESM.jpg]
