# Supplementary material for: The role of tyrosine hydroxylase–dopamine pathway in Parkinson’s disease pathogenesis
Source: Cell Mol Life Sci. 2022 Nov 21;79(12):599. doi: 10.1007/s00018-022-04574-x (PMC9678997; doi:10.1007/s00018-022-04574-x)
Supplement: Supplementary file 10 — Supplementary file10 (DOCX 14 KB) [file 18_2022_4574_MOESM10_ESM.docx]

**Supplementary Table 1. LRRK2 and PINK1 cloning primers**

| **Name of primers** | **Sequence of primers** |
| --- | --- |
| LRRK2 pcDNA3.1(-) infusion XbaI forward primer | AAACGGGCCCTCTAGATGGCTAGTGGCAGCTGT |
| LRRK2 pcDNA3.1(-) infusion HindIII reverse primer | GTTTAAACTTAAGCTTTACTCAACAGATGTTCGTCTCA |
| PINK1 pcDNA3.1(-) infusion XbaI forward primer | AAACGGGCCCTCTAGATGGCGGTGCGACAGGCG |
| PINK1 pcDNA3.1(-) infusion HindIII reverse primer | GTTTAAACTTAAGCTTCACAGGGCTGCCCTCCATGAGC |
| LRRK2 infusion NotI forward primer | ACAGATCTTGCGGCCATGGCTAGTGGCAGCTGT |
| LRRK2 infusion XbaI reverse primer | ACAAAGATCCTCTAGGAGATCCTCTTCCGAAATAAGCTT |
