# Supplementary material for: The role of tyrosine hydroxylase–dopamine pathway in Parkinson’s disease pathogenesis
Source: Cell Mol Life Sci. 2022 Nov 21;79(12):599. doi: 10.1007/s00018-022-04574-x (PMC9678997; doi:10.1007/s00018-022-04574-x)
Supplement: Supplementary file 11 — Supplementary file11 (DOCX 13 KB) [file 18_2022_4574_MOESM11_ESM.docx]

**Supplementary Table 2. LRRK2 and PINK1 mutagenesis primers**

| **Name of primers** | **Sequence of primers** |
| --- | --- |
| LRRK2 K1906A forward primer | GAAGTGGCTGTGGCGATTTTTAATAAACATACATCACTCAGGC |
| LRRK2 K1096A reverse primer | GCCTGAGTGATGTATGTTTATTAAAAATCGCCACAGCCACTTC |
| LRRK2 D1994A forward primer | GCCATGATTATATACCGAGCCCTGAAACCCCAC |
| LRRK2 D1994A reverse primers | GTGGGGTTTCAGGGCTCGGTATATAATCATGGC |
| LRRK2 D2017A forward primer | CCATCATTGCAAAGATTGCTGCCTACGGCATTGC |
| LRRK2 D2017A reverse primer | GCAATGCCGTAGGCAGCAATCTTTGCAATGATGG |
| LRRK2 G2019S forward primer | GCAAAGATTGCTGACTACAGCATTGCTCAGTACTGC |
| LRRK2 G2019S reverse primer | GCAGTACTGAGCAATGCTGTAGTCAGCAATCTTTGC |
| PINK1 A339T forward primer | CCCCCGCCTCACCGCCATGATGCTGCT |
| PINK1 A339T reverse primer | AGCAGCATCATGGCGGTGAGGCGGGGG |
| PINK1 E231G forward primer | GGTTCCTCCAGCGGAGCCATCTTGAACACA |
| PINK1 E231G reverse primer | TGTGTTCAAGATGGCTCCGCTGGAGGAACC |
