# Supplementary material for: The role of tyrosine hydroxylase–dopamine pathway in Parkinson’s disease pathogenesis
Source: Cell Mol Life Sci. 2022 Nov 21;79(12):599. doi: 10.1007/s00018-022-04574-x (PMC9678997; doi:10.1007/s00018-022-04574-x)
Supplement: Supplementary file 12 — Supplementary file12 (DOCX 13 KB) [file 18_2022_4574_MOESM12_ESM.docx]

**Supplementary Table 3. LRRK2 and PINK1 sequencing primers**

| **Name of primers** | **Sequence of primers** |
| --- | --- |
| LRRK2 sequencing primer 420 | TCTCCTCCTAAC |
| LRRK2 sequencing primer 826 | AGGCTTACATTAGG |
| LRRK2 sequencing primer 1511 | CGCTTCGAGCTA |
| LRRK2 sequencing primer 2317 | AAAGCGTTGACG |
| LRRK2 sequencing primer 2992 | GATGCCCTAAGC |
| LRRK2 sequencing primer 3592 | TTGCGGTCTTTA |
| LRRK2 sequencing primer 4183 | TTTGCAGGTCGTG |
| LRRK2 sequencing primer 4606 | TCGGAGCGTAAA |
| LRRK2 sequencing primer 5069 | TTATCATCCGACTA |
| LRRK2 sequencing primer 5585 | CTGACCTGCCTAG |
| LRRK2 sequencing primer 6110 | GGTTTCGTGC |
| LRRK2 sequencing primer 6649 | TCTGGGACAC |
| LRRK2 sequencing primer 7105 | AATAGCCCTGTT |
| PINK1 sequencing primer 423 | CCCGTTGGACACGAGACGCTTG |
| PINK1 sequencing primer 1003 | AGCCCCCGCCTCGCC |
| PINK1 sequencing primer 1238 | AGACAGCCGTTTCCGCCCCGAT |
