# Supplementary material for: The role of tyrosine hydroxylase–dopamine pathway in Parkinson’s disease pathogenesis
Source: Cell Mol Life Sci. 2022 Nov 21;79(12):599. doi: 10.1007/s00018-022-04574-x (PMC9678997; doi:10.1007/s00018-022-04574-x)
Supplement: Supplementary file 13 — Supplementary file13 (DOCX 13 KB) [file 18_2022_4574_MOESM13_ESM.docx]

**Supplementary Table 4. Quantitative real time RT-PCR primers**

| **Name of primers** | **Sequence of primers** |
| --- | --- |
| Human TH forward primer | gccctaccaagaccagacgta |
| Human TH reverse primer | cgtgaggcatagctcctga |
| Mice TH forward primer | CCAAGGTTCATTGGACGGC |
| Mice TH reverse primer | CTCTCCTCGAATACCACAGCC |
| Human PINK1 forward primer | GGCCTCATCGAGGAAAAACAG |
| Human PINK1 reverse primer | GGACTGCCCTATCAGATACTCC |
| Human LRRK2 forward primer | GCTGCTATGCCTTTCTTGCC |
| Human LRRK2 reverse primer | AGATTTCCAGTGTGCGGGAC |
