# Supplementary material for: The role of tyrosine hydroxylase–dopamine pathway in Parkinson’s disease pathogenesis
Source: Cell Mol Life Sci. 2022 Nov 21;79(12):599. doi: 10.1007/s00018-022-04574-x (PMC9678997; doi:10.1007/s00018-022-04574-x)
Supplement: Supplementary file 14 — Supplementary file14 (DOCX 12 KB) [file 18_2022_4574_MOESM14_ESM.docx]

| **Supplementary Table 5. List of primary antibodies** | | | | |
| --- | --- | --- | --- | --- |
| **Antibody** | **Host species** | **Dilution** | **Company** | **Cat. No.** |
| Nanog | Rabbit | 1:200 | ProteinTech | 14295-1-AP |
| OCT4 | Mouse | 1:500 | ProteinTech | 60242-1-Ig |
| SOX2 | Mouse | 1:125 | R&D Systems | MAB2018 |
| TRA-1-60R | Mouse | 1:100 | STEMCELL Technologies | 60064 |
| HSP60  TH  TH  Activated caspase 3  Tubulin | Rabbit  Mouse  Rabbit  Rabbit  Mouse | 1:200  1:100  1:500  1:400  1:500 | ProteinTech  Santa Cruz  Sigma  Cell Signaling  Sigma | 66041-1-Ig  Sc-25269  T8700  9661  T5076 |
|  |  |  |  |  |
