# Supplementary material for: The role of tyrosine hydroxylase–dopamine pathway in Parkinson’s disease pathogenesis
Source: Cell Mol Life Sci. 2022 Nov 21;79(12):599. doi: 10.1007/s00018-022-04574-x (PMC9678997; doi:10.1007/s00018-022-04574-x)
Supplement: Supplementary file 15 — Supplementary file14 (DOCX 12 KB) [file 18_2022_4574_MOESM15_ESM.docx]

| **Supplementary Table 6. List of secondary antibodies** | | | | |
| --- | --- | --- | --- | --- |
| Alexa Fluor 488 anti-mouse IgG | Goat | 1:500 | Thermo Fisher Scientific | A-11001 |
| Alexa Fluor 594 anti-rabbit IgG | Goat | 1:500 | Thermo Fisher Scientific | A-11012 |
