# Supplementary material for: The role of tyrosine hydroxylase–dopamine pathway in Parkinson’s disease pathogenesis
Source: Cell Mol Life Sci. 2022 Nov 21;79(12):599. doi: 10.1007/s00018-022-04574-x (PMC9678997; doi:10.1007/s00018-022-04574-x)
Supplement: Supplementary file 16 — Supplementary file14 (DOCX 18 KB) [file 18_2022_4574_MOESM16_ESM.docx]

**Supplementary Methods**

**Human ESC lines**

The H9 human ESC were originally obtained from WiCell Research Institute (Madison, WI). human ESC lines were maintained in mTeSR1 media (Stemcell Technologies) under feeder-free conditions on Matrigel (BD) coated plates at 37 °C in a humidified CO2 incubator. Cells were routinely passaged at 70 - 80 % confluency using EDTA (0.5 mM). All hPSC lines were confirmed negative for mycoplasma contamination and displayed normal karyotypes.

**Three germ layer differentiation assay of iPSCs**

Induced iPSCs were differentiated to ectoderm, mesoderm and endoderm using the STEMdiff™ Trilineage Differentiation Kit (STEMCELL Technologies) according to the manufacturer's protocol. Briefly, iPSCs were dissociated using TrypLE (Invitrogen) and the single cell suspension was plated into Matrigel coated six well plate in TeSR™-E8 medium (STEMCELL Technologies) for 24 hr. Cells were then feed daily with endoderm or mesoderm differentiation media for 5 days or ectoderm differentiation media for 7 days respectively. The differentiated cells were assessed by immunofluorescence using the Human Three Germ Layer 3-Color Immunocytochemistry Kit (R&D Systems).

**Generation of hTH overexpressing ESC line**

The cDNA sequence of human TH (CCDS7731.1) was cloned into a lentiviral backbone (Addgene, #27150) to generate 3rd generation dox-inducible lentiviral particles. To prepare for the transduction, hESCs were dissociated with TrypLETM Express to single cells and plated into Matrigel-coated cell culture plates in mTeSR1 media supplemented with ROCK inhibitor. On the following day, cells were transduced with the lentiviral vector expressing hTH and the lentiviral expression vector of rtTA (Addgene, #20342). In the next day, culture media was completely replaced with mTeSR1 medium, which was used until the end of the experiments. Cells were selected with appropriate antibiotics from day 3 to 7 to enrich the transduced cells. Dox-dependent overexpression of hTH was validated by western blotting.

**Immunochemistry**

Cultured cells or hMLOs slides on glass cover slip were fixed in 4% paraformaldehyde for 30 min at room temperature. After washing the cells with PBS for 3 times, cells were permeabilized and blocked with 5 % bovine serum albumin (BSA) in PBS containing 0.3 % Triton-X 100 for 1 hr. After blocking, cells were incubated overnight with primary antibodies at 4 °C. Subsequently cells were washed with PBS for 3 times and 5 min each, followed by 1-hour incubation with respective secondary antibodies at 1: 500 dilution in PBS. After washing cells with PBS, cells on glass cover slips were mounted using mounting medium containing 4,6-diamidino-2-phenylindole (DAPI). The immunofluorescence images were taken using Zeiss LSM 710 Upright microscope. Details of primary and secondary antibodies used are listed in following tables.

**TG *Drosophila* lines**

Transgenic UAS-hDJ-1 7-1(III) and UAS-hDJ-1 5-3(III) lines to overexpress human WT DJ-1 as well as UAS-dLRRK IR 1-1/TM6B line to knockdown *Drosophila* LRRK2 are gifts from Prof Lu Bing Wei (Stanford University. USA). Transgenic UAS-DJ1a/cyo and UAS-DJ1b lines to overexpress *Drosophila* DJ-1a and DJ-1b as well as DJ-1b[delta93] (knockout DJ-1b), DJ-1a[delta72]/cyo (knockout DJ-1a) and DJ1-DKO delta72; delta93/Tb (knockout both DJ-1a and DJ-1b) lines are gifts from Prof Nancy Bonini (Pennsylvania University. USA).
